# Genomic Insights Into the Pathogenicity of a Novel Biofilm-Forming *Enterococcus* sp. Bacteria (*Enterococcus lacertideformus*) Identified in Reptiles

**DOI:** 10.3389/fmicb.2021.635208

**Published:** 2021-03-02

**Authors:** Jessica Esther Agius, David Norton Phalen, Karrie Rose, John-Sebastian Eden

**Affiliations:** ^1^Faculty of Science, Sydney School of Veterinary Science, University of Sydney, Camden, NSW, Australia; ^2^Schubot Exotic Bird Health Center, College of Veterinary Medicine & Biomedical Sciences, Texas A&M University, College Station, TX, United States; ^3^Australian Registry of Wildlife Health, Taronga Conservation Society Australia, Mosman, NSW, Australia; ^4^Centre for Virus Research, The Westmead Institute for Medical Research, Westmead, NSW, Australia; ^5^Marie Bashir Institute for Infectious Diseases and Biosecurity, Faculty of Medicine and Health, Sydney School of Medicine, University of Sydney, Camperdown, NSW, Australia

**Keywords:** antimicrobial resistance, bacteria, biofilm, Christmas Island, enterococcus, reptiles, virulence, whole genome sequencing

## Abstract

Whole genome analysis of a novel species of enterococci, *Enterococcus lacertideformus*, causing multi-systemic and invariably fatal disease in critically endangered Christmas Island reptiles was undertaken to determine the genetic elements and potential mechanisms conferring its pathogenic nature, biofilm-forming capabilities, immune recognition avoidance, and inability to grow *in vitro.* Comparative genomic analyses with related and clinically significant enterococci were further undertaken to infer the evolutionary history of the bacterium and identify genes both novel and absent. The genome had a G + C content of 35.1%, consisted of a circular chromosome, no plasmids, and was 2,419,934 bp in length (2,321 genes, 47 tRNAs, and 13 rRNAs). Multi-locus sequence typing (MLST), and single nucleotide polymorphism (SNP) analysis of multiple *E. lacertideformus* samples revealed they were effectively indistinguishable from one another and highly clonal. *E. lacertideformus* was found to be located within the *Enterococcus faecium* species clade and was closely related to *Enterococcus villorum* F1129D based on 16S rDNA and MLST house-keeping gene analysis. Antimicrobial resistance (DfreE, EfrB, tetM, bcrRABD, and sat4) and virulence genes (Fss3 and ClpP), and genes conferring tolerance to metals and biocides (*n* = 9) were identified. The detection of relatively few genes encoding antimicrobial resistance and virulence indicates that this bacterium may have had no exposure to recently developed and clinically significant antibiotics. Genes potentially imparting beneficial functional properties were identified, including prophages, insertion elements, integrative conjugative elements, and genomic islands. Functional CRISPR-Cas arrays, and a defective prophage region were identified in the genome. The study also revealed many genomic loci unique to *E. lacertideformus* which contained genes enriched in cell wall/membrane/envelop biogenesis, and carbohydrate metabolism and transport functionality. This finding and the detection of putative enterococcal biofilm determinants (EfaAfs, srtC, and scm) may underpin the novel biofilm phenotype observed for this bacterium. Comparative analysis of *E. lacertideformus* with phylogenetically related and clinically significant enterococci (*E. villorum* F1129D, *Enterococcus hirae* R17, *E. faecium* AUS0085, and *Enterococcus faecalis* OG1RF) revealed an absence of genes (*n* = 54) in *E. lacertideformus*, that encode metabolic functionality, which potentially hinders nutrient acquisition and/or utilization by the bacterium and precludes growth *in vitro*. These data provide genetic insights into the previously determined phenotype and pathogenic nature of the bacterium.

## Introduction

Emerging infectious diseases are increasingly impacting reptile populations globally and pose a significant threat to their conservation and biodiversity (Cabañes et al., 2014; Jacobson et al., 2014; O’Dea et al., 2016; Tetzlaff et al., 2017). An example of a pathogen that is posing a major threat to multiple reptilian species is a novel bacterial species; *Enterococcus lacertideformus.* This novel pathogen is the only known species of *Enterococcus* that acts as a primary pathogen and is not associated with hospital-acquired infections. This organism poses a major threat to the captive breeding colonies of the Extinct in the Wild Christmas Island Lister’s geckos (*Lepidodactylus listeri*) and blue-tailed skinks (*Cryptoblepharus egeriae*), where it has breached quarantine measures causing mortality events in conservation breeding colonies on Christmas Island (Rose et al., 2017). The origin is unknown, but this bacterium appears to have established itself either in the environment or in host species on Christmas Island where it is regularly observed to cause disease in the invasive free-ranging Asian house (*Hemidactylus frenatus*) and mute (*Gehyra mutilata*) geckos. Although at the time of the initial outbreak *E. lacertideformus* was thought to be a locally occurring disease, research suggests that the incident may not be isolated. Outbreaks likely caused by *E. lacertideformus* or a similar organism with identical morphology, lesion type and distribution have been described in Singapore house geckos from Malaysia (McNamara et al., 1994), four species of captive lizards (Carolina anole, Cape girdled lizards, Balkan green lizards, and European green lizards) held in a Polish collection (Zwart and Cornelisse, 1972), and in free-ranging brown anoles (*Anolis sagrei*) from Florida, United States of America (Ossiboff et al., 2020). Molecular analyses further undertaken on the brown anoles revealed that a 1,400 bp segment of the 16s rRNA gene was 100% identical to *E. lacertideformus* (Ossiboff et al., 2020).

Infection with *E. lacertideformus* is believed to result from bite wounds to the face or by colonization of the oral cavity followed by tissue invasion. Lizards present initially with gingival swelling and the formation of gelatinous subcutaneous nodules of the face and head. With time, the disease becomes systemic and expansile nodular lesions are seen in multiple tissues. Microscopically, chains of bacterial cocci are surrounded by a thick biofilm-like matrix comprising pilus extensions radiating from the cell wall. The large colonies of bacteria replace bone and soft tissues in the head, and compress and replace the normal parenchyma of the other tissues that they invade. The biofilm surrounding *E. lacertideformus* appears to mask it from the host’s immune system as histologically limited inflammatory responses are observed surrounding the bacteria (Rose et al., 2017).

As efforts to culture *E. lacertideformus in vitro* have been unsuccessful, no information on antimicrobial susceptibilities, virulence traits and metabolic traits exist. This lack of understanding regarding the bacterium’s inability to grow *in vitro*, in addition to its novelty, unique structure and apparent evasion of host immune responses highlights the need to study its biology through *in silico* investigations into the organism’s genome and evolutionary origins. This foundational genomic work will contribute to fulfilling the overarching goals of diagnostic tool development, prognostication, treatment, and biosecurity risk analyses and practice.

## Materials and Methods

### Sample Collection and Preparation

Tissue samples were collected from three wild Asian house geckos on Christmas Island showing signs consistent with infection with *E. lacertideformus* (Rose et al., 2017). Two geckos (samples: 10706.1 and 10706.10) were collected from southeast of Christmas Island in September 2017 (South Point, GPS coordinates: 10°33′42.05′′S 105°38′55.07′′E), and one gecko (sample: 10702.133) was collected from the centerof Christmas Island (Pink House Research Station, GPS coordinates: 10°29′30.50′′S 105°38′49.60′′E) in May 2018. The affected geckos were euthanised with an overdose of alfaxalone (Alfaxane, Jurox Animal Health). The diseased tissues from the mucosa and submucosa of the maxilla were aseptically collected and stored in 90% ethanol until DNA extraction.

### DNA Isolation

Genomic DNA was isolated from the alcohol-fixed tissues using a modified animal tissue protocol and the DNeasy Blood and Tissue Kit (Qiagen, Victoria, Australia). Briefly, tissues were rehydrated with four 1× phosphate buffered saline washes to remove residual fixative, mechanically ground and pre-treated with a lysozyme digestion step (25 mM Tris-HCl pH 8, 2.5 mM EDTA, 1.2% triton X-100, 20 mg μL^–1^ lysozyme) recommended for lysis of Gram-positive bacteria. Samples were then digested with proteinase K for 6 h and the DNA purified following the manufacturer’s instructions.

### Whole Genome Sequencing

Purified genomic DNA was prepared as shotgun libraries using the Truseq DNA PCR-free library and sequenced on the Illumina NovaSeq 6000 platform generating at least 150 Gb of 150 bp paired-end reads per library at the Australian Genome Research Facility (AGRF).

### Sequence Assembly and Analysis

The raw sequence reads were assessed for quality using FastQC (Andrews, 2010). The raw sequence reads were quality trimmed using Trimmomatic (Bolger et al., 2014), sequences with a Phred score less than 25 were removed. To obtain an initial estimate of sequencing coverage of *E. lacertideformus* present in each library, the trimmed reads were mapped against the *Enterococcus hirae* strain R17 genome (NCBI GenBank accession CP015516) (Peng et al., 2017) using the Burrow-Wheeler Aligner (BWA-MEM v0.7.12) with default settings (Li and Durbin, 2009). This revealed that sample 10702.133 contained the highest coverage depth (mean 1006.52×) in comparison to samples 10706.1 and 10706.10, with mean coverages of 245.72× and 8.73×, respectively. Given the greater sequencing coverage, 10702.133 was therefore chosen as the sample to represent the genome of *E. lacertideformus* in our study. To perform genome assembly, trimmed reads were first mapped to the reference assembly *Gekko japonicus* v1.1 (NCBI genome assembly GCF_001447785.1) using BWA-MEM v0.7.12 to remove host sequences. The unmapped, i.e., ‘non-Gecko’ reads from sample 10702.133 were then *de novo* assembled using both SPAdes v3.13.0 (Bankevich et al., 2012) and MEGAHIT v1.1.3 (Li et al., 2015), the remaining samples 10706.1 and 10706.10 were assembled using only MEGAHIT v1.1.3. Default parameters were used for each genome assembler method, except for setting the distribution of kmers to be 21, 29, 39, 59, 79, 99, 119, and 127. Both SPAdes and MEGAHIT were evaluated to determine the method that produced the assembly with the highest quality. The non-gecko contigs were then aligned against the NCBI non-redundant nucleotide and protein databases with an *E*-value threshold of 1e-10 using BLAST (Altschul et al., 1990) and DIAMOND (Buchfink et al., 2015), respectively. The contigs were then filtered by taxonomic group (‘Bacteria’ or ‘Enterococcus’), sequence coverage depth as determined by expected coverage from initial mapping to *E. hirae* R17 genome, and contig length sequence (>250 bp). The *de novo* assemblies using SPAdes and MEGAHIT were then examined by QUAST v5.0.2 (Gurevich et al., 2013), using default parameters with *E. hirae* R17 as a reference genome. Based on the QUAST results, there were negligible differences in the quality of the MEGAHIT and SPAdes assemblies for sample 10702.133, furthermore, both assemblies were structurally similar. However, following initial annotations, MEGAHIT in comparison to the SPAdes assembly comprised a greater number of CDS and was able to resolve expected rRNA genes, therefore was used to represent the whole genome shotgun assembly of *E. lacertideformus.*

### Genome Annotation

Gene identification and annotation was performed by the DFAST prokaryotic genome annotation pipeline v1.2.4^1^ with default settings (Tanizawa et al., 2018). ABRicate v0.8 (Seemann, 2020) PointFinder (Zankari et al., 2017) was used to screen the 10702.133 MEGAHIT contigs for putative resistance genes and virulence factors using multiple databases – Antibiotic Resistance Gene-ANNOTation (ARG-ANNOT) (Gupta et al., 2014), ResFinder (Zankari et al., 2012), Resistance Gene Identifier (RGI) (Alcock et al., 2020), Comprehensive Antibiotic Resistance Database (CARD) (Jia et al., 2017), PlasmidFinder (Carattoli et al., 2014), NCBI AMRFinderPlus (Feldgarden et al., 2019), and Virulence Factor Database (VFDB) (Chen et al., 2016). Positive identification of resistance genes and virulence factors were indicated by a *E*-value threshold of 1e-100, and a minimum coverage and nucleotide identity of 75 and 85%, respectively. The Antibacterial Biocide and Metal Resistance Genes Database (BacMet) v2.0 for experimentally confirmed (*n* = 753) and predicted (*n* = 155,512) resistance genes were downloaded from the BacMet website^2^ to predict antibacterial biocide and metal resistance encoding genes (Pal et al., 2014) and used for annotation with DIAMOND v3.2.10 using the BLASTx algorithm and an *E*-value threshold 1e-100. The presence of CRISPR loci were predicted using the CRISPERFinder (Grissa et al., 2007) tool^3^ and CRISPI^4^ with default settings (Rousseau et al., 2009). The abundance and diversity of insertional elements and transposons were identified using ISfinder^5^ (Siguier et al., 2006) and BLASTn v2.2.31+, with an E-value threshold of 1e-50. Discovery and annotation of prophage loci within the genome was undertaken using PHASTER^6^ (Arndt et al., 2016) and BLASTn v2.2.31+. The genome was additionally investigated for integrative conjugative elements (ICEs) by homology searches using web nucleotide BLAST against 714 ICEs downloaded from the ICEberg database v2.0 (Liu et al., 2019). Only complete ICE sequences were included with *E*-value and bit-score thresholds of 1e-150 and 400, respectively. The genome was additionally mined for secondary metabolite biosynthetic gene clusters using antiSMASH bacterial v5.0^7^ with default settings (Blin et al., 2019), and ribosomally synthesized and post-translationally modified peptides and bacteriocins using BAGEL4^8^ (van Heel et al., 2018). Horizontal gene transfer was detected by the genomic island tool Islandviewer 4, using the prediction method IslandPath-DIMOB (sequence composition method)^9^ (Bertelli et al., 2017). The whole genome shotgun assembly of *E. lacertideformus* was ordered against the reference genome *E. hirae* R17 using the Mauve Contig Mover (MCM) (Rissman et al., 2009). *Enterococcus hirae* R17 (accession: NZCP015516) was chosen as the reference genome as multiple alignments with other comparator enterococci (*E. villorum* F1129D, accession: BJWF01000000, *E. faecium* AUS0085, accession: CP006620, and *E. faecalis* OG1RF, accession: NC017316) using the MCM algorithm revealed that the *E. hirae* R17 alignment had the lowest number of locally collinear blocks, rearrangements, and inversions. However, as a complete genome of *E. lacertideformus* is not available, genomic rearrangements of this bacterium cannot be excluded. Proteins classified as hypothetical by the program were confirmed by BLASTp and renamed if they had *E*-value and percent identity thresholds of 1e-50 and 80, respectively. The query data and functions of all non-ribosomal hypothetical proteins identified using BLASTp were also listed. Repeat sequences were identified using Tandem Repeats Finder Program v4.09^10^ with default parameters (Benson, 1999).

### Biofilm and Pili Virulence Factors

Nucleotide sequences of well-defined genes involved in biofilm development and pili expression among enterococci were identified and extracted from known enterococcal biofilm-forming and clinically significant genomes *E. faecalis* OG1RF, *E. faecalis* LN68, *E. faecalis* KUB3006 and *E. faecium* DO. The nucleotide sequences of enterococcal surface protein (esp), endocarditis and biofilm-associated pili genetic locus (EbpA, EbpB, and EbpC), *E. faecalis* endocarditis-associated antigen A (EfaA), aggregation substance (Agg), adhesion of collagen of *E. faecalis* (ace), adhesion to collagen from *E. faecium* (acm), second collagen adhesin of *E. faecium* (scm), nidogen-binding LPXTG surface adhesion (sgrA), sortase-encoding gene (srt), hyaluronidases (hylA and hylB), gelatinase (gelE), serine protease (sprE), and fecal streptococci regulator locus (fsrA, fsrB, and fsrC) were manually queried using BLASTn on Geneious Prime v2020.0.5^11^ to determine if *E. lacertideformus* contained any homologous regions (*E*-value threshold 1e-10).

### Comparative Analysis

The trimmed non-Gecko reads from samples 10702.133 (accession: SRX9763078), 10706.10 (accession: SRX9763079), and 10706.1 (accession: SRX9763080) were aligned to our reference *E. lacertideformus* whole genome shotgun assembly (MEGAHIT assembly of sample 10702.133) (accession: JADAKE000000000) using BBMap v37.98 (Bushnell, 2014) and the resultant BAM alignments were visualized in Geneious Prime v2020.0.5. Sample 10702.133 contained the highest coverage depth (mean 2478.26×) in comparison to samples 10706.1 and 10706.10, with mean coverages of 523.34× and 13.47×, respectively. The samples were screened for single nucleotide polymorphisms (SNPs) with a minimum coverage of 10×. A nucleotide was identified as a putative SNP if it occurred in more than 50% of the read coverage. Each SNP was manually inspected to confirm the alignment and coverage. Pairwise identity percentages and the number of SNPs between each sample and the reference were calculated to determine the clonality between samples and if mixed organisms were present. To verify clonality, seven house-keeping multi-locus sequence typing (MLST) genes (atpA, adk, ddl, gdh, gyd, pstS, and purK) from 10702.133, 10706.1 and 10706.100 were identified, extracted, concatenated, and aligned using MAFFT aligner v7.450, and the number of SNPs counted.

A comparative analysis of the *E. lacertideformus* whole genome shotgun assembly to four other complete genomes (*E. villorum* F1129D, *E. hirae* R17, *E. faecium* AUS0085, and *E. faecalis* OG1RF) was made using the CGView Comparison Tool (CCT) (Stothard et al., 2019). The contigs of *E. lacertideformus* were ordered against the *E. hirae* R17 genome with MCM. This tool was additionally used to assign genes of *E. lacertideformus* to Clusters of Orthologous Groups (COGs), and generate circular genomic maps containing genome features (G + C content, G + C skew, CDS, rRNA, and tRNA). A complete count of the genes for each COG functional feature common among all five genomes were determined, and their proportions comparatively illustrated in a circle chart. Functional features where *E. lacertideformus* contained the largest number of genes when compared to comparator genomes were extracted and illustrated in a bar chart. The number of COGs present across all five genomes (Core COGs) and the number of COGs occurring only in *E. lacertideformus* (Specific COGs) were defined. To determine ‘Core COGs,’COG source IDs shared between *E. lacertideformus* and all four-comparator genomes *E. hirae, E. villorum, E. faecium*, and *E. faecalis* for each feature were counted. When COG source IDs were present only in *E. lacertideformus* and absent in all comparator genomes these were considered ‘Specific COGs’ and were summed for each feature. The number of individual genes for each feature for both the core and specific COGs were summed and tabulated as in some instances multiple genes were associated with a single COG ID. Additionally, COG source IDs present among all comparator genomes, but absent in *E. lacertideformus* were defined.

The *E. lacertideformus* genome was aligned to reference genomes *E. villorum* F1129D, *E. hirae* R17, and *E. faecium* AUS0085 using the progressiveMauve algorithm (Darling et al., 2010). *Enterococcus faecalis* OG1RF was not used as a comparator due to its genomic dissimilarity to *E. lacertideformus*, and inability to adequately resolve novel regions. Alignments were visualized in the Mauve Genome Viewer using Geneious Prime v2020.0.5 to identify regions unique to *E. lacertideformus*. True deletions on comparison were unable to be resolved as they may represent missing assembly data. Regions were illustrated as insertions when they were absent in at least two of the three comparator genomes and had a length of greater than 5,000 nucleotides. Insertions which fulfilled these criteria were not included if all the genes of that region were categorized as having a hypothetical or unknown function. Genes possibly explaining the biofilm phenotype of *E. lacertideformus*, categorized into the [M] functional category (cell wall/membrane/envelope biogenesis), were further labeled and their function and query statistics tabulated. Operons for the [M] category genes identified in the unique regions of *E. lacertideformus* were predicted using POEM py3k (Xiao, 2019).

### Phylogenetic Analysis

The complete 16S rDNA gene from *E. lacertideformus* was aligned against 32 reference sequences of known *Enterococcus* and outgroup bacteria downloaded from NCBI GenBank. The reference sequences selected represented a suitable diversity of enterococcal species, particularly focusing on the *E. faecium* clade as preliminary phylogenetic analyses revealed *E. lacertideformus* mapped to this species group (Rose et al., 2017). All sequences were aligned using MAFFT aligner v7.450 with the FFT-NS-i × 1,000 algorithm (Katoh and Standley, 2013) in Geneious Prime v2020.0.5. The General-Time Reversible model (GTR) with Gamma distributed rate classes (*n* = 4) including Invariant sites (GTR + G + I) was found to be the best-fit substitution model according to Bayesian Information Criterion (BIC) in MEGA-X v10.1.1 (Kumar et al., 2018). The 16S rDNA phylogenetic tree was inferred using the maximum likelihood approach in PhyML v20150402 with the GTR + G + I model and 1,000 bootstrap replicates (Guindon et al., 2005). The phylogeny represented the best topology with nearest-neighbor interchange (NNI) and sub-tree pruning and re-grafting (SPR) searches. The phylogeny was visualized using Figtree v1.4.4^12^. The phylogram was rooted using an outgroup, the branch leading to *Vagococcus penaei* (strain: CD276) and *Vagococcus martis* (strain: D7T301) and was shown with bootstrap replicates hidden when less than 50%. In addition to the 16S rDNA phylogeny, the MLST allelic profiles of *E. faecium* house-keeping genes were downloaded from pubMLST^13^ and were identified and extracted from the *E. lacertideformus* assembly as previously stated. The complete sequences of the seven house-keeping genes (atpA, adk, ddl, gdh, gyd, pstS, and purK) were queried against the reference sequences of known enterococcus and outgroup bacteria used in the 16S rDNA phylogeny and extracted. The seven constitutive genes for each bacterium were then concatenated to produce a multi-locus alignment, and the nucleotide sequences aligned as before. Phylogenetic analysis of this dataset was performed employing the GTR + G + I model (against best model by BIC) with 1,000 bootstrap replicates. The phylogram generated from this approach was visualized and rooted as the 16S rDNA phylogeny.

## Results

### Features of the Genome

**FIGURE 1:**
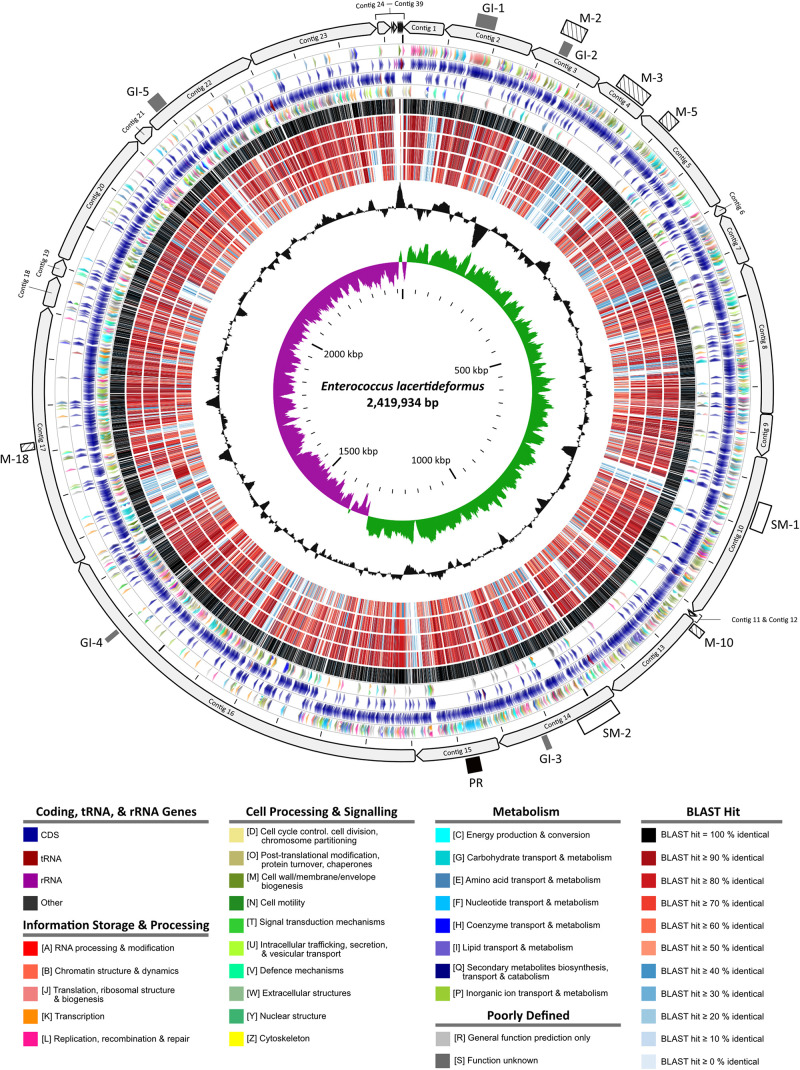
Circular map of the *Enterococcus lacertideformus* genome. A graphical circular map of the incomplete *E. lacertideformus* genome performed with the CGview comparison tool (Stothard et al., 2019). Contigs were ordered against the *E. hirae* R17 genome using Mauve Contig Mover. Concentric rings from outside to inside are as follows: (1) contigs of the *E. lacertideformus* incomplete genome (2) Scale marks of the *E. lacertideformus* genome. The gray rectangles represent genomic islands (GI-1 to G1-5), white-filled rectangles represent secondary metabolite gene cluster regions (SM-1 to SM-2), hatched boxes represent COG feature [M – cell wall/membrane/envelope biogenesis] dominant regions unique to *E. lacertideformus*, and the black rectangle represents a prophage region (PR). (3) COG features for protein coding genes on the forward strand. (4) Locations of protein coding, tRNA, and rRNA genes on the forward strand. (5) Locations of protein coding, tRNA, and rRNA genes on the reverse strand. (6) COG features for protein coding genes on the reverse strand. Gene colors indicate protein coding, tRNA, rRNA and COG features to which they belong are shown in the key below the map. (7–11) Regions of similarity detected using BLASTp (*E*-value threshold = 0.1) between CDS translations shared by *E. lacertideformus*, and those of reference genomes *E. villorum* F1129D, *E. hirae* R17, *E. faecium* AUS0085, and *E. faecalis* OG1RF, respectively. Regions of similarity are colored (black to blue) based on the percent identity between the aligned sequence segments are shown below the map. (12) The black plot depicts GC content with the peaks extending toward the outside of the circle representing GC content above the genome average, whereas those extending toward the center mark segments with GC content lower than the genome average. (13) The innermost plot depicts GC skew. Both base composition plots were generated using a sliding window of 10,000 nt.

Maxilla tissue DNA from three *E. lacertideformus* infected Asian House geckos were subjected to whole genome sequencing on the Illumina NovaSeq platform producing 498,507,015 to 833,901,565 paired reads across all the samples. The relative abundance of *E. lacertideformus* in each tissue sample varied, with sample 10702.133 containing the highest coverage depth (mean 1006.52×) in comparison to samples 10706.1 and 10706.10, with means coverages of 245.72× and 8.73×, respectively. Given the higher abundance of *E. lacertideformus* in the sequenced libraries, host depleted sequence reads from sample 10702.133 was used to generate our reference genome by *de novo* assembly. A comparison of assembly methods using both SPAdes and MEGAHIT revealed negligible differences in the overall assembly quality according to N50 and L50 metrics. However, more genes and expected rRNA were identified using the MEGAHIT assembly contig set, therefore was used as our final draft genome and more subsequent genome annotations and comparisons. The initial MEGAHIT assembly of sample 10702.133 produced a total of 3,367,650 non-Gecko contigs of which 829,116 returned any BLAST result less than 1e-5. The majority of contigs (*n* = 827,464/829,116) were host derived (Eukaryota) and likely due to differences in our study species (*H. frenatus*) and the reference genome used for host DNA removal (*G. japonicus*). Importantly, of the non-Eukaryota contigs, 139 were annotated as ‘Bacteria,’ and 46 specifically as ‘*Enterococcus*.’ Furthermore, 39 ‘Enterococcus’ contigs remained following removal of sequences less than 250 bp in length (Supplementary Table 1). The 39 contigs made up the final contig set of the draft *E. lacertideformus* genome (accession: JADAKE000000000) and ranged between 269 and 431,263 bp in length with an N50 and L50 of 137,263 and 6, respectively, and a total length of 2,419,934 bp. The G + C content of the draft genome was 35.1% and contained a total of 2,321 genes (2,257 CDS), 47 tRNAs, 13 rRNAs (*n* = 4 5S rRNA, *n* = 3 16S rRNA, *n* = 6 23S rRNA), and 4 ncRNAs (Figure 1). No plasmids were identified in the assembly. The BLASTp results illustrated by rings 6 – 10 in Figure 1 indicate that the proteins of *E. lacertideformus* do not demonstrate significant homology to any of the comparator genomes (*E. villorum, E. hirae, E. faecium*, and *E. faecalis*), particularly *E. faecalis* (innermost BLAST ring).

### Antimicrobial Resistance and Virulence Genes

The whole genome shotgun assembly of *E. lacertideformus* was screened using ABRicate and PointFinder to identify antimicrobial resistance and virulence encoding genes. Of the eight databases used, only CARD and VFDB returned hits. A total of two virulence factors, including the ATP-dependant Clp protease proteolytic subunit (ClpP) and fibrinogen binding MSCRAMM (Fss3) were identified with VFDB, and two antimicrobial resistance genes, including dihydrofolate reductase (DfrE) and multidrug efflux ABC transporter subunit (EfrB) were identified with CARD (Table 1). Genes and gene-products mediating intrinsic resistance to cephalosporins (CroRS, *IreK*, and *IreP*), penicillin (PBPs, *pbp5*), low-level aminoglycosides [AAC(6′)-Ii, APH(3′)-IIIa] and clindamycin (*lsa*) were not identified when screening *E. lacertideformus*. Antibacterial biocide and metal resistance genes were additionally predicted (Table 1). Putative resistance genes related to magnesium, copper, zinc, cadmium, cobalt, tellurite, selenite and chlorhexidine were encoded in the genome.

**TABLE 1:** Virulence determinants, antimicrobial resistance, and biocide and metal resistance genes present in the *E. lacertideformus* genome.

**Gene**	**Database**	**Product**	**Function**	**Nucleotide positions**	***E*-value**	**Identity (%)**	**Query coverage (%)**
ClpP	VFDB	ATP-dependent Clp protease proteolytic subunit ClpP	Hydrolysis of proteins to small peptiles/degradation of misfolded proteins	Contig 9 660936–661529	9e-141	99.5	100.0
Fss3	VFDB	Fibrinogen binding MSCRAMM	Binding to collagen type V and fibrinogen	Contig 17 1684933–1685949	0.0	95.0	100.0
DfrE	CARD	Dihydrofolate reductase	Resistance to trimethoprim	Contig 14 1056463–1056963	2e-109	88.5	99.0
EfrB	CARD	Multidrug efflux ABC transporter subunit EfrB	Efflux pump conferring resistance to macrolide, rifamycin and fluoroquinolone	Contig 9 658166–659182	0.0	95.3	100.0
mgtA	EC	Magnesium-transporting ATPase, P-type 1	Mediates magnesium influx to the cytosol	Contig 8 595875–598523	0.0	100.0	100.0
copB	EC	Copper-exporting P-type ATPase B	Required for the copper-inducible expression of copper resistance	Contig 17 1713336–1713836	1.0e-103	91.6	100.0
copA	EC	Copper-importing P-type ATPase A	Copper import under copper limiting conditions	Contig 17 1716144–1717397	0.0	86.1	99.0
ziaA	EC	Zinc-transporting ATPase	Zinc-transporting ATPase ziaA	Contig 17 1721452–1723533	0.0	100.0	100.0
cadA/yvgW	EC	Cadmium, zinc and cobalt-transporting ATPase	Couples the hydrolysis of ATP with the transport of cadmium, zinc and cobalt out of the cell	Contig 17 1716144–1717397	0.0	86.1	99.0
recG	EC	ATP-dependent DNA helicase RecG	Can confer resistance to tellurite and selenite	Contig 2 112412–114448	0.0	100.0	100.0
tcrB	EC	Cation transport ATPase (P-type)	The transferable, plasmid-localized copper resistance efflux ATPase	Contig 17 1713858–1714784	1.0e-178	86.6	96.0
chtR	EC	Cognate DNA-binding response regulator	Chlorhexidine tolerance response regulator	Contig 20 2035895–2036569	5.0e-162	100.0	100.0
mgtA	PR	Magnesium-translocating P-type ATPase	Magnesium transmembrane transporter activity	Contig 8 595875–598523	0.0	100.0	100.0

*CARD, comprehensive antibiotic resistance database; EC, experimentally confirmed resistance database; VFDB, virulence factor database; PR, predicted resistance database*.

### CRISPR Genes

Using the CRISPRCasFinder tool the genome of *E. lacertideformus* was identified to have three CRISPR elements and two Cas clusters (Table 2). One Cas cluster (CAS-TypeIIA) was flanked by three CRISPR-Cas genes, consisting of *cas1*, *cas2* and *csn2*. The second Cas cluster was identified upstream of Cas-TypeIIA and contained only the *cas1* and *cas2* genes.

**TABLE 2:** CRISPR-Cas elements present in the *E. lacertideformus* assembly.

**Gene**	**DR consensus**	**DR length**	**Spacer sequence**	**Spacers count**	**CRISPR length**	**CRISPR positions**
CRISPR 1	TTTTTTTTTTTTTTTTTTTTTTTTT	25	TTTTTTTTTTTTTTGATCTA	1	69	Contig 3/4 216238–216307
CRISPR 2	CTGTTTTATCAGAAAAGGTGCAAT	24	TAGCAGAAAAGCAACCAGAATCGGAAGACAAGA	1	80	Contig 9 646250–646330
CRISPR 3	AATTTTTCTTCATTTCCTAGTATCT	25	TGTTTACTTTATCTAACAATTGTATGTTTTTAGCTAATTCA	1	90	Contig 23 2380171–2380261
**Gene**	**Product**	**Number of copies**	**Gene status/system**	***E*-value**	**Query/identity %**	**Nucleotide positions**
*cas1*	CRISPR associated endonuclease	2	Accessory/general-class 1 Accessory/general-class 2	1.1e-180 4e-158	100.0/84.3 100.0/85.4	Contig 8 576,235–576,975 Contig 22 2,203,903–2,204,763
*cas2*	CRISPR-associated endoribonuclease	2	Accessory/general-class 1 Accessory/general-class 2	3e-61 6e-68	100.0/86.4 100.0/94.4	Contig 8 577,029–577,352 Contig 22 2,203,559–2,303,870
*csn2*	type II-A CRISPR-associated protein	1	Mandatory/general-class 2	7e-98	96.0/74.0	Contig 22 2,202,913–2,203,581

*DR, direct repeat.*

### Mobile Genetic Elements

The *E. lacertideformus* assembly was investigated for mobile genetic elements, including ICEs, prophages, and transposable elements. The genome was identified to contain 36 ICEs exhibiting homology to six ICE families (Tn5801, Tn916, Tn5253, ICESt1, ICESa2603, and unclassified) (Supplementary Table 2). Each of the classified ICE families, excluding ICESt1, harbored tetracycline resistance from *Staphylococcus aureus* (Tn5801 and Tn916), *Streptococcus suis* (ICESa2603), *Streptococcus pneumoniae* (Tn5253 and Tn916), *Streptococcus gallolyticus* (Tn916), *Streptococcus pseudintermedius* (Tn916), *Streptococcus agalactiae* (Tn916), and *Filifactor alocis* (Tn916). Additional resistance profiles for cadmium and arsenic (ICEa2603) from *Streptococcus dysgalactiae*, streptothricin (ICESsu05SC260) from *Streptococcus suis*, and bacitracin resistance (Tn916) from *Clostridium perfringens* were identified.

The *E. lacertideformus* assembly was additionally screened for prophages using PHASTER, which revealed no intact or questionable prophage regions, however, a single incomplete prophage region (PHASTER score < 70) 14.8 kb in length, at position 1,125,949 to 1,140,766 bp, and containing a total of 21 proteins (11 phage hits and 10 hypothetical protein hits) was identified (Supplementary Table 3). Seven phage-like proteins remained following filtering with an *E*-value threshold of 1e-10. These prophages were from Siphoviridae and Myoviridae, with Myoviridae being the most prevalent viral family.

Insertion elements were additionally identified in *E. lacertideformus* using ISfinder, and included ISEfa10, ISEfa5, and ISEfa11 (Supplementary Table 4). The IS elements identified were small (13–43% of the total IS element), therefore, their functions and contribution to the genome plasticity and resistance capabilities of *E. lacertideformus* cannot be reliably inferred.

### Genomic Islands

Five regions (GI 1 to 5) totalling 51,927 bp in length (2.15% of the genome) were predicted as genomic islands (GI) in the *E. lacertideformus* assembly (Supplementary Table 5). All five GIs were distinctly separate from one another and were therefore not suspected of being a single GI. Each of the five GIs encoded both hypothetical proteins and proteins with known functionality (Supplementary Table 5). Independent identification of hypothetical proteins using BLASTp revealed that out of 44 proteins identified as hypothetical by the Islandviewer program, 18 returned a positive BLAST result (including 2 ribosomal proteins) (Supplementary Table 6). Of these, six genes frequently encoded on GIs were identified, and included three recombinase family proteins (GI-1 and GI-5), a ISL3 family transposase (GI-3), a site-specific recombinase, phage integrase family (GI-4), and a tyrosine-type recombinase/integrease (GI-4). Unlike GIs 2 to 5, GI 1 was observed to contain several housekeeping genes, and therefore this region may not in fact represent a true GI.

### Secondary Metabolite Gene Clusters

The *E. lacertideformus* assembly contained two secondary metabolite regions (Figure 1). The first region (SM-1) comprised of two types, the Linear azol(in)e-containing peptides (LAP), and thiopeptide cluster. Both clusters spanned a total of 29,133 bp (genomic positions 717,698–746,830 bp). The LAP/thiopeptide region contained four core biosynthetic genes (YcaO × 2, lantibiotic biosynthesis dehydratase C-term, SagB), three additional biosynthetic genes (crotonyl-CoA reductase/alcohol dehydrogenase × 2, phosphopantothenoylcysteine decarboxylase), five transport-related genes (ABC transporter ATP-binding protein × 2, ABC transporter permease protein × 2, ABC transporter related protein), and a single regulatory gene (MarR family transcriptional regulator). The second region (SM-2) spanning 41,158 bp (976,525–1,017,682 bp) contained Type III polyketide synthase (T3PKS). The T3PKS region contained one core biosynthetic gene (hydroxymethylglutaryl-CoA synthase), five additional biosynthetic genes (acetyl-CoA acetyltransferase, hydroxymethylglutaryl-CoA reductase, aldo/keto reductase, GTP-binding protein LepA, and alpha/beta hydrolase fold protein), one transport-related gene (ABC transporter ATP-binding protein), and a single regulatory gene (GntR family transcriptional regulator). No areas of interest were discovered when *E. lacertideformus* was mined for RiPPs and bacteriocins using BAGEL4.

### Tandem Repeats

A total of 118 tandem repeats (TRs) were identified in the *E. lacertideformus* assembly with period sizes ranging from to 1 to 285 bp. The total TR length and percentage of genome coverage for period size were 4,049 bp and 0.167%, respectively. Many of the TRs identified in *E. lacertideformus* were minisatellites (10–100 bp), with 56% of all repeats located in protein-coding regions (Supplementary Table 7).

### Biofilm-Associated Genotypes

Screening *E. lacertideformus* for genotypes encoding biofilm formation and pili expression revealed three putative genes with an *E*-value threshold of 1e-10 and percentage identical sites >60%. The genes EfaAfs (2,054,498–2,055,305 bp) (*E. faecalis* KUB3006), srtC (1,634,471–1,634,826 bp) (*E. faecalis* OG1RF), and scm (702,666–702, 835 bp) (*E. faecium* DO) returned an *E*-value and percent identity of 3.77e-73 and 68.7%; 1.08e-16 and 66.3%; and 1.16e-22 and 75.1%, respectively.

### Phylogenetic Analysis

**FIGURE 2:**
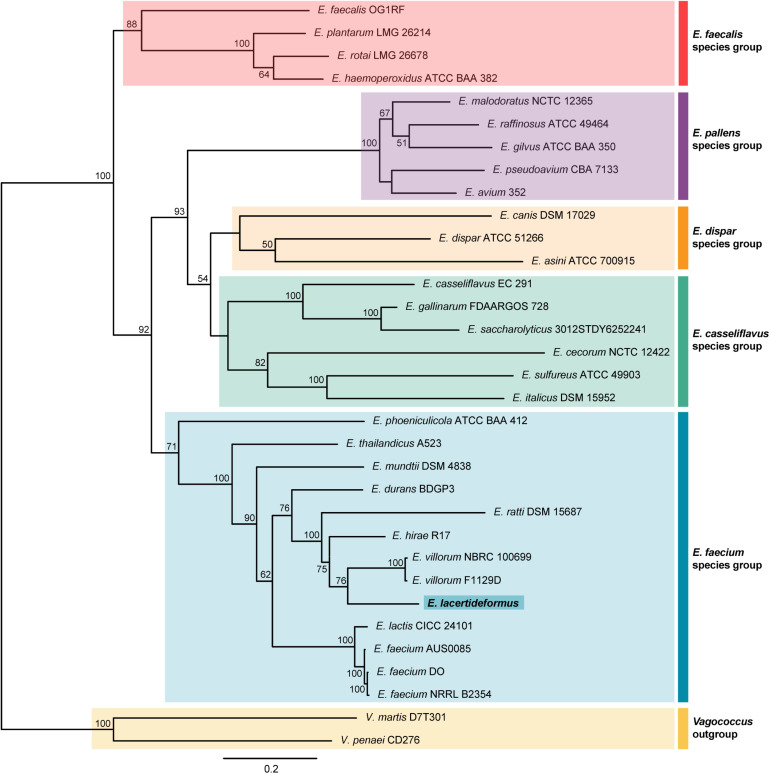
Multi-locus phylogenetic tree. The evolutionary history using seven enterococcal house-keeping genes (atpA, adk, ddl, gdh, gyd, pstS, and purK) was inferred by the Maximum Likelihood method, employing the General-Time-Reversible model with Gamma distributed plus Invariant sites (GTR + G + I), with 33 nucleotide sequences including the novel *E. lacertideformus*. The percentage of trees in which the associated taxa clustered together is shown next to the branches and is derived from 1,000 bootstraps (bootstraps > 50% shown). There was a total of 3,461 positions in the final dataset. The various enterococcus clades are shaded, with the *E. faecium* species group highlighted in blue, and novel *E. lacertideformus* denoted in bold (dark blue shading).

The entire 16S rDNA sequence of *E. lacertideformus* was compared with the 16S rDNA sequences of other members of the *Enterococcus* genus (*n* = 30), along with two strains from the genus *Vagococcus* (*L. penaei*, *V. martis*) that were used as outgroups (Supplementary Table 8). The maximum likelihood phylogeny placed *E. lacertideformus* within the *E. faecium* clade (Supplementary Figure 1), clustering with both *E. villorum* strains (F1129D and NBRC 100699). The maximum sequence identity of *E. lacertideformus* 16S rDNA to the reference sequences included in the phylogram was 99.39% (*E. villorum* F1129D). The phylogeny additionally showed that the *E. lacertideformus* and *E. villorum* cluster were closely related to *Enterococcus mundtii*, but distant from *Enterococcus durans* and *E. faecium* strains. However, the evolutionary relationships of *E. lacertideformus* to members of the *Enterococcus* genus could not be adequately defined using 16S rDNA sequences due to poor clustering support. The multi-locus phylogenetic tree provided a more reliable estimate of the evolutionary relationships across enterococci, dividing all 31 strains of *Enterococcus* into five distinct species groups (*E. faecalis*, *Enterococcus pallens*, *Enterococcus dispar*, *Enterococcus casseliflavus*, and *E. faecium*) (Holzapfel and Wood, 2014; Zhong et al., 2017). Indeed, the multi-locus phylogeny estimated using the seven concatenated house-keeping genes, in agreement with the 16S rDNA phylogeny, shows that *E. lacertideformus* is a member of the *E. faecium* species group, and is a sister species to both *E. villorum* strains (Figure 2). The *E. lacertideformus* and *E. villorum* cluster were closely related to *E. hirae*, but distant from *E. mundtii* and *E. faecium* strains. The bootstrap support for the multi-locus phylogeny was significantly improved on comparison to the 16S rDNA phylogeny, with all nodes in the *E. faecium* clade providing support greater than 60 percent.

### Comparative Genomics

Pairwise comparisons and MLST analysis of house-keeping genes between the *E. lacertideformus* assembly (sample 10702.133) and the other samples – 10706.10 and 10706.1 – indicated that all three individual samples were highly clonal and likely represented a dominant strain. Re-mapping the sequence reads from sample 10702.133 back onto the 10702.133-derived *E. lacertideformus* assembly revealed a total of 14 SNPs present likely from assembly errors. Samples 10706.10 and 10706.1 showed a greater but not dissimilar number of SNPs when compared to the *E. lacertideformus* assembly, including 20 and 35 SNPs, respectively (Table 3). Furthermore, an analysis of the key MLST genes revealed no SNPs between the three samples.

**TABLE 3:** Pairwise comparisons (SNPs and nucleotide similarity percentage) of three *E. lacertideformus* sample reads to the final *E. lacertideformus* assembly (sample: 10702.133).

	**10702.133**	**10706.10**	**10706.1**
***E. lacertideformus* assembly (sample: 10702.133)**	SNPs = 14 99.9994%	SNPs = 20 99.9992%	SNPs = 35 99.9986%

**FIGURE 3:**
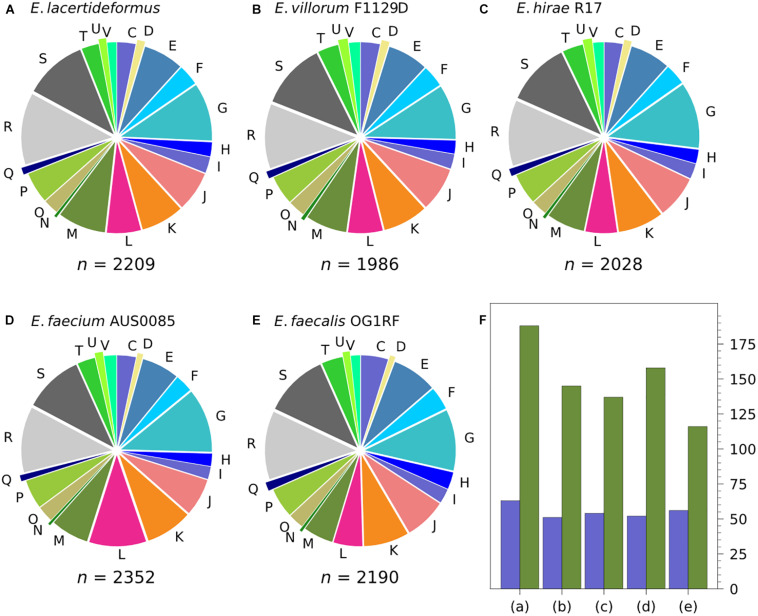
Classification of COGs in *E. lacertideformus* and comparator genomes by feature categories. The proportions of COGs by features common across **(A)** *E. lacertideformus*, **(B)** *E. villorum* F1129D, **(C)** *E. hirae* R17, **(D)** *E. faecium* AUS0085, and **(E)** *E. faecalis* OG1RF are illustrated. A total count of these common COGs for each genome are shown below the respective chart. One letter abbreviations for the features: [C], energy production and conversion; [D], cell cycle control, cell division, chromosome partitioning; [E], amino acid transport and metabolism; [F], nucleotide transport and metabolism; [G], carbohydrate transport and metabolism; [H], coenzyme transport and metabolism; [I], lipid transport and metabolism; [J], translation, ribosomal structure and biogenesis; [K], transcription; [L], replication, recombination and repair; [M], cell wall/membrane/envelope biogenesis; [N], cell motility; [O], posttranslational modification, protein turnover, chaperones; [P], inorganic ion transport and metabolism; [Q], secondary metabolites biosynthesis, transport and catabolism; [R], general function prediction only; [S], function unknown; [T], signal transduction mechanisms; [U], intracellular trafficking, secretion and vesicular transport; [V], defense mechanisms. The bar chart **(F)** illustrates the number of COGs (*y*-axis) for the [M] (green) and [I] (purple) functional groups which showed the largest disparity between **(A)** *E. lacertideformus*, and comparator genomes **(B)** *E. villorum*, **(C)** *E. hirae*, **(D)** *E. faecium*, and **(E)** *E. faecalis*.

The COGs of *E. lacertideformus* were classified into 20 features. A significant majority of them were assigned to well-defined functional features; however, the two single largest categories were represented by functionally uncharacterized COGs (categories [R] general function prediction, and [S] function unknown) (Figure 3A). Other significant proportions of COGs included [G] carbohydrate transport and metabolism, [M] cell wall/membrane/envelope biogenesis, and [K] transcription. The functional features [Z] cytoskeleton and [N] cell motility represented the minority of COGs. The COG features and their proportions common across *E. lacertideformus* and the comparator genomes *E. villorum* F1129D, *E. hirae* R17, *E. faecium* AUS0085, and *E. faecalis* OG1RF were illustrated (Figures 3A–E). Comparative genomic analysis of COG features revealed that *E. lacertideformus* contained 29.7, 37.2, 19.0, and 62.1% more genes encoding cell wall/membrane/envelope biogenesis [M]; and 23.5, 16.7, 21.2, and 12.5% encoding lipid transport and metabolism [I] when assessed against comparator genomes *E. villorum* F1129D, *E. hirae* R17, *E. faecium* AUS0085 and *E. faecalis* OG1RF, respectively (Figure 3).

The number of core and *E. lacertideformus-*specific individual COGs for a particular feature totalled 1,022 (1923 genes) and 27 (37 genes), respectively (Table 4). Core COGs with a functional prediction were assigned to 18 of 23 COG features, and the number of COGs (*n* = 347, 44.5%) and genes (*n* = 704, 47.1%) for class metabolism were most prevalent in comparison to the remaining functional classes: cell processing and signaling (COGs, *n* = 165, 21.1%; genes, *n* = 361, 24.1%), and information, storage, and processing (*n* = 269, 34.4%; genes, *n* = 430, 28.8%). Of the core metabolic features, carbohydrate transport and metabolism had the greatest number of COGs (*n* = 72). Specific COGs unique to *E. lacertideformus* and with a functional prediction were identified across 9 features (Table 4), and were mainly associated with class metabolism (COGs, *n* = 13, 48.1%; genes, *n* = 20, 62.5%), particularly for features [C] energy production and conversion (COGs, *n* = 3; genes, *n* = 4), [P] inorganic ion transport and metabolism (COGs, *n* = 3; genes, *n* = 5), and [G] carbohydrate transport and metabolism (COGs, *n* = 2; genes, *n* = 5) (Supplementary Table 9).

**TABLE 4:** Features of core and strain-specific COGs present in the *E. lacertideformus* assembly.

**Function of each feature**	**Core COGs**	**Specific COGs**	**Function**	**Core COGs**	**Specific COGs**
[A] RNA processing and modification	0	0	[Y] Nuclear structure	0	0
[B] Chromatin structure and dynamics	0	0	[Z] Cytoskeleton	0	0
[J] Translation, ribosomal structure and biogenesis	130 (156)	0	[C] Energy production and conversion	40 (67)	3 (4)
[K] Transcription	64 (163)	0	[G] Carbohydrate transport and metabolism	72 (183)	2 (5)
[L] Replication, recombination and repair	75 (111)	1 (1)	[E] Amino acid transport and metabolism	65 (143)	2 (2)
[D] Cell cycle control, cell division, chromosome partitioning	13 (26)	0	[F] Nucleotide transport and metabolism	52 (76)	0
[O] Posttranslational modification, protein turnover, chaperones	37 (55)	0	[H] Coenzyme transport and metabolism	28 (43)	0
[M] Cell wall/membrane/envelope biogenesis	56 (150)	2 (3)	[I] Lipid transport and metabolism	32 (57)	2 (3)
[N] Cell motility	4 (9)	0	[Q] Secondary metabolites biosynthesis, transport and catabolism	9 (23)	1 (1)
[T] Signal transduction mechanisms	29 (61)	1 (1)	[P] Inorganic ion transport and metabolism	49 (112)	3 (5)
[U] Intracellular trafficking, secretion and vesicular transport	18 (24)	0	[R] General function prediction only	111 (237)	5 (5)
[V] Defense mechanisms	8 (36)	0	[S] Function unknown	130 (191)	5 (7)
[W] Extracellular structures	0	0	**Total**	**1022 (1923)**	**27 (37)**

The number of individual COGs for a particular feature absent from *E. lacertideformus* but present in all comparator enterococci totalled 54 (50 individual genes) (Supplementary Table 10). Of the 54 COGs, 37 were annotated with a functional prediction, and the remaining 17 as function unknown or general function prediction. The majority of COGs with a functional characterization (*n* = 27, 73.0%) occurred in features representing metabolism ([C] = 5, [E] = 10, [F] = 3, [G] = 6, [H] = 1, [P] = 2), particularly iron (flavodoxin/ferredoxin oxidoreductase, ferredoxin, and ferrous transport systems) and sugar-related metabolism (trehalose and maltose hydrolase, maltose binding periplasmic protein, and ABC-type maltose transport system permease component).

Counts for ‘Core COGs’ represent the number of individual COG IDs for each feature only when they were present in all the five enterococcus genomes examined (*E. lacertideformus, E. villorum* F1129D, *E. hirae* R17, *E. faecium* AUS0085, and *E. faecalis* OG1RF). Counts for the ‘Specific COGs’ indicate the number of COG IDs identified only in the *E. lacertideformus* assembly, and not in the other enterococcal comparator genomes examined.

The number of core COGs listed for each feature do not represent the total number of core COGs as some counts were not considered because particular COG IDs did not occur across all five genomes.

Bracketed values in the ‘Core COGs’ and ‘Specific COGs’ columns indicate the number of genes identified for each COG feature in *E. lacertideformus*.

### Regions Unique to *E. lacertideformus*

**FIGURE 4:**
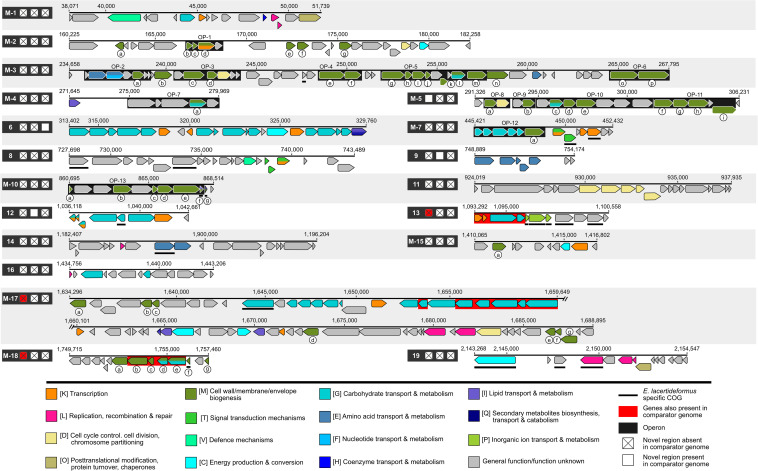
Regions unique to *E. lacertideformus.* The unique regions present in the *E. lacertideformus* whole genome scaffold and absent in at least two of the comparator genomes *E. villorum, E. hirae* and *E. faecium* are illustrated. Individual regions unique to *E. lacertideformus* are numbered (1–19) according to their order in the genome. Boxes adjacent to numbered regions indicate the presence and/or absence of the specific region in comparator genomes. Boxes from left to right refer to *E. villorum* F1129D, *E. hirae* R17 and *E. faecium* AUS0085, respectively. Boxes marked [X] indicate that the novel region displayed is absent in a particular comparator genome. Genes are colored according to their classification into different COG functional categories. Genes encoding the cell wall/membrane/envelope biogenesis [M] feature are alphabetically labeled for further reference to tabulated data describing gene functionality (Supplementary Table 11). Clusters of genes within a unique region highlighted in red refer to genes also present in a particular comparator genome (check box also marked red). Numbers above the genes indicate nucleotide positions.

Comparison of the *E. lacertideformus* assembly to *E. villorum* F1199D, *E. hirae* R17 and *E. faecium* AUS0085 revealed a total of 19 unique regions greater than 5,000 bp in length present in the *E. lacertideformus* genome and absent in at least two of the three comparator genomes (Figure 4). Of the 19 regions, the entire length of 12 insertions were absent in all comparator genomes (box marked with *X*), an additional three were partially absent across all comparators (red shading), and four were present in a single comparator genome only (box shaded with white). Genes with [M] cell wall/membrane/envelope functionality were most prominent across all unique regions (green shading) (*n* = 56), followed by genes with [G] carbohydrate transport and metabolism functionality (turquoise shading) (*n* = 49). The 56 genes with cell wall/membrane/envelope biogenesis were identified in nine of the 19 unique regions (M-2, M-3, M-4, M-5, M-7, M-10, M-15, M-17, and M-18). Regions completely unique to *E. lacertideformus* and containing [M] functionality were identified in regions M-2, M-3, and M-10, regions M-5 and M-18 were classified into this category but also occurred in a single comparator enterococcus. All [M] category genes across unique regions one to 19 were queried and the majority exhibited similarity to enterococcal organisms, with their sequence identity ranging from 36.8 to 95.4% (mean 72.9%) (Supplementary Table 11). A total of 13 operons (black shading) were predicted for [M] category genes in the unique regions of *E. lacertideformus and* belonged to M-2 (*n* = 1), M-3 (*n* = 5), M-4 (*n* = 1), M-5 (*n* = 4), M-7 (*n* = 1), and M-10 (*n* = 1) (Figure 4). Hypothetical proteins and proteins with carbohydrate transport and metabolism functionality were also commonly identified within these predicted operons (Figure 4).

## Discussion

The emergence of this multi-systemic and fatal bacterium prompted a thorough metagenomics investigation to gain insights into the genomic content of *E. lacertideformus*, and to investigate the genetic basis underpinning its unique biofilm phenotype, pathogenic nature, and inability to grow *in vitro*. Comparative genomics were further undertaken to understand the evolutionary history of *E. lacertideformus.*

### The Evolutionary History of *Enterococcus lacertideformus* and Its Clonality

The MLST phylogeny of concatenated house-keeping genes provided stronger discriminatory power, and the evolutionary relationships among enterococci were more consistent with the topology of robust phylogenies and previous studies (Holzapfel and Wood, 2014). The MLST phylogeny divided the enterococci into five distinct lineages: *E. faecalis, pallens*, *dispar, casseliflavus*, and *faecium*, and placed *E. lacertideformus* in a monophyletic cluster within the *E. faecium* clade, and a sister species to both *E. villorum* strains. As expected, the phylogenetic tree based on the 16S rDNA gene provided poor reliability (low bootstrap support) and delineation of enterococci at the species-level, as has been previously demonstrated (Rose et al., 2017; Zhong et al., 2017).

The MLST results and low number of SNPs identified across the *E. lacertideformus* assemblies indicated that the sequences were indistinguishable, and therefore appear to represent a clonal expansion of a specific *E. lacertideformus* strain, a trait characteristic of highly pathogenic enterococci (Homan et al., 2002; Ruiz-Garbajosa et al., 2006). The variant frequency for several of these SNPs approached the 50% cut-off, which is likely due to base calling errors in sequencing, or the presence of a mixed sample population. Therefore, each sample analyzed may in effect contain fewer SNPs than reported, subsequently the number of SNPs was considered negligible. The presence of non-target organisms within the assemblies was considered. *E. lacertideformus* was suggested to be the dominant variant because of the lack of SNPs identified, the use of the aseptic sample collection technique, the absence of enterococcal growth *in vitro*, along with cytological and histological evidence of only *E. lacertideformus* organisms (Rose et al., 2017). However, as sequencing was not performed on a pure culture, this assembly should be considered as a group rather than a single strain.

### Comparative Genomics Highlights Genes Linked to Cell Wall and Biofilm Formation and Novel Metabolism

Enriched COGs assigned to the features cell wall/membrane/envelope biogenesis [M] and lipid transport and metabolism [I] in *E. lacertideformus* relative to comparator enterococci indicate a strong selective pressure and niche selection for these genes. The enriched COGs for [I] suggests that lipid utilization capacity in *E. lacertideformus* is extensive relative to comparator enterococci. Lipids are critical elements of bacterial cell walls and membranes, and are responsible for biomembrane synthesis, cell membrane physical and chemical properties (Bogdanov et al., 2002), and modulating biofilm formation of Gram-positive bacteria *in vivo* (Theilacker et al., 2009). The physiological associations between these features, and the biofilm phenotype and pathogenic nature of *E. lacertideformus* supports the functional enrichment of [M] and [I] features. Further investigation into the functions of the enriched genes/gene pathways will aid in understanding their importance in contributing to the fitness of *E. lacertideformus.*

The high prevalence of core and *E. lacertideformus*-specific COGs for class metabolism, particularly carbohydrate transport and metabolism functionality [G] suggests an extensive carbohydrate utilization capacity by *E. lacertideformus*. Carbohydrate transport and metabolism plays a pivotal role in biofilm formation of Gram-positive bacteria, particularly in the clinically significant *E. faecalis* and *E. faecium* (Pillai et al., 2004). Each of the carbohydrate genes specific to *E. lacertideformus* encoded various subunits of sugar phosphotransferase system (PTS) transporters. Sugar phosphotransferase system transporters are involved in signal transduction and in the transport and metabolism of sugars, all essential for modulating biofilm formation and EPS synthesis (Loo et al., 2003; Lazazzera, 2010; Kawada-Matsuo et al., 2016; Heo et al., 2019). An additional *E. lacertideformus*-specific COG; glycerol-3-phosphate cytidylyltransferase (COG0615) may further correlate with the biofilm-forming capacity of this bacterium. Four genes encoding this enzyme were identified in features [M] (*n* = 2) and [I] (*n* = 2). Glycerol-3-phosphate cytidylyltransferase is involved in the biosynthesis of teichoic acid linkage units, which are important for cell wall biogenesis in Gram-positive bacteria (Rodrigues et al., 2016). Therefore, the identification of these *E. lacertideformus*-specific genes may explain its unique biofilm phenotype and should be considered as pertinent candidates for future experimental tests. Further work incorporating transcriptomic analyses of these targets, particularly glycerol-3-phosphate should be undertaken in concert with molecular cloning experiments. Molecular cloning of target genes expressed by the bacterium into strains of non-biofilm-forming, phylogenetically related enterococci will provide insights into their precise function, and whether they contribute to the biofilm phenotype of *E. lacertideformus.*

Several unique regions containing genes with [M] functionality occurred in predicted operons and were frequently clustered with genes of unknown function. Considering enterococcal biofilm formation is often encoded by complementary, overlapping and potentially redundant gene clusters (e.g., *bee* locus, locus and *fsr* locus) (Hashem et al., 2017), the proteins containing domains of unknown function identified in *E. lacertideformus* may be involved in biofilm formation as a result of their loci. Additionally, several uncharacterized enterococcal loci are involved or expressed during the development of biofilms (Ballering et al., 2009) in animal models of infection (Frank et al., 2015), further suggesting their potential role in enterococcal biofilm production. The identification of these regions, particularly those predominating in [M] category genes, and their occurrence in multiple distinct operons are likely responsible for the distinctively thick matrix and fibrillar capsular projections surrounding this bacterium and its immune evasion strategies. As this research identified core and *E. lacertideformus*-specific genes based on COG IDs, future studies will be required to incorporate more reliable methods to identify COGs at a gene-level basis.

### Genomics of *Enterococcus lacertideformus* Pathogenesis

Virulence factors encoding for structural elements including pili and the capacity to form biofilms have been identified across several species of enterococci (Ch’ng et al., 2019). The molecular mechanisms that promote or inhibit this complex process (Ch’ng et al., 2019) are not well characterized (Hashem et al., 2017), with nearly one quarter of the 100 genetic loci involved in enterococcal biofilm formation encoding products annotated as hypothetical or of unknown function (Willett et al., 2019). The three putative virulence determinants identified in *E. lacertideformus* are also commonly found in clinical *E. faecium* and *E. faecalis* isolates. The EfaAfs adhesin is presumed to play a pivotal role in the pathogenesis of endocarditis and adherence of enterococci to biotic and abiotic surfaces (Lowe et al., 1995; Singh et al., 1998), scm has been shown to mediate binding to collagen type V and fibrinogen (Sillanpää et al., 2008), and srtC (or bps, biofilm- and pilus- associated sortase) has been shown to play a role in pili biogenesis (Sillanpää et al., 2013). The genes EfaAfs and scm therefore potentially play a role in bacterial colonization of the host, while srtC is possibly linked to the distinct extracellular matrix produced by *E. lacertideformus*. Although enterococcal genotype-phenotype correlations have not been well established, the genetic determinants agg, gelE, and the fsr locus (notably fsrA and fsrB) are strong predictors of enterococcal biofilm formation (Hashem et al., 2017). Agg, gelE and fsr genes among others were not identified in *E. lacertideformus*, however, given its novelty and discernible biofilm phenotype, further determinants encoding biofilm formation are likely present, but do not confer sufficient homology to known genotypes for effective identification.

The occurrence of virulence factors in enterococci other than *E. faecium* and *E. faecalis* is either rare or does not occur (Beukers et al., 2017), thus identification of the ATP-dependent Clp protease proteolytic subunit (ClpP); responsible for the adaptation to multiple stressors via degradation of misfolded and accumulating proteins (Michel et al., 2006), and the fibrinogen binding MSCRAMM (Fss3); responsible for binding to host fibrinogen and collagen to initiate infection (Sillanpää et al., 2009), was unexpected. With little information currently known on the nature of virulence genes in enterococcal species other than *E. faecium* and *E. faecalis* (Beukers et al., 2017), additional, previously uncharacterised virulence genes may occur in *E. lacertideformus.*

The transcriptional regulatory protein LytR identified in GI-2 may also play an important role in the virulence of *E. lacertideformus.* The LytR protein is responsible for regulating genes involved in autolysis, apoptosis, biofilm formation and cell wall metabolism (Chatfield et al., 2005). This island is additionally located in the novel unique region M-2 (160,225–182,258bp) of *E. lacertideformus* (Figure 4), which is comprised primarily of genes encoding the [M] feature. Therefore, the location of GI-2 and its presence on a predicted GI indicates that it was likely horizontally acquired. Genes encoding restriction endonuclease and methyltransferase (aka restriction-modification system) (Furuta et al., 2011) were identified in GI-5. Restriction-modification systems function as a defense system against invading DNA elements (e.g., bacteriophages), and are involved in the generation of genetic diversity (Vasu and Nagaraja, 2013). Additionally, genes encoding transposase, integrase, recombinase, or transferase functionality were identified across each of the GIs of *E. lacertideformus*, indicating that these discrete DNA segments were likely acquired through horizontal gene transfer and have the potential to promote evolutionary beneficial and pathogenic traits (Juhas et al., 2009).

The number of TRs identified in *E. lacertideformus* (*n* = 118) are similar to the closely related *E. hirae* R17 (*n* = 127). However, only 70 TRs were identified in two pathogenic strains of *E. faecium* (strain 140623 and SBS-1) (Gan et al., 2020). Many of the TRs identified in *E. lacertideformus*, like in *E. hirae* R17, and both *E. faecium* strains were minisatellites (10–100 bp) located in protein-coding regions, indicating their potential role in targeted gene variation. Therefore, the large number of TRs identified in *E. lacertideformus* in comparison to the pathogenic *E. faecium* strains, in addition to their location in the genome, suggests that *E. lacertideformus* may rely on TRs to regulate the activity of genes needed to adapt to environmental stressors.

### *Enterococcus lacertideformus* Contains Few Genes Associated With Antimicrobial Resistance

Based on genomic data, *E. lacertideformus* is only resistant to trimethoprim (DfrE), bacitracin (bcrRABD), tetracycline (tetM), and streptothricin (sat4). The multidrug efflux ABC transporter subunit EfrB, a component of the EfrAB efflux pump that confers resistance to fluoroquinolones, macrolides and rifamycins was detected. However, the multidrug efflux ABC transporter also requires the EfrA subunit to be functional (Lee et al., 2003), and this sequence was not identified. The limited antibiotic resistance profile observed in *E. lacertideformus* suggests that it is likely not driven by exposure to recently developed and clinically significant antibiotics (Martínez, 2008).

Although *E. lacertideformus* may be susceptible to a wide range of antimicrobials, the true intrinsic antimicrobial resistance pattern of this organism can only be inferred using genomics. Several species of enterococci exhibiting phenotypic resistance to numerous antimicrobials analyzed in a comparative genomics study produced no significant findings on ResFinder or CARD (Beukers et al., 2017), highlighting the limitations of these databases. Other factors may also determine the susceptibility of *E. lacertideformus* to antibiotics. Studies have shown that biofilm formation increases the minimum inhibitory concentrations of antibiotics up to 1,000 times (Mah, 2012); thus *E. lacertideformus* may be protected against antimicrobials, rendering it difficult to eradicate.

Given that the antibiotic susceptibility of *E. lacertideformus* can only be predicted using genomics, it will be necessary to do transcriptomics or undertake *in vivo* treatment trials in naturally or experimentally infected reptiles to determine the bacterium’s true sensitivity to antibiotics. Based on the genomic findings, enrofloxacin would be the first treatment choice of *E. lacertideformus* as it is a bactericidal, broad-spectrum fluoroquinolone (Schroder, 1989) that exhibits biofilm-inhibiting properties (Yang et al., 2017) and is widely used in reptile medicine because of its high therapeutic index, and favorable pharmacokinetic profile (Salvadori and Vito, 2015). This antibiotic has additionally been shown to achieve plasma concentrations that may be effective at treating *E. lacertideformus* and comparable pathogens with an enrofloxacin MIC ≤ 0.5 μg/mL *in vitro* (Agius et al., 2020). The antibiotics amoxicillin clavulanic acid, rifampicin, and clarithromycin are also bactericidal in nature, exhibit biofilm inhibiting or penetrating properties, and are likely effective against *E. lacertideformus* based on the absence of associated resistance encoding genes in this genetic data.

### CRISPR-Cas Arrays in *Enterococcus lacertideformus* May Account for the Absence of Plasmids

The identification of functional CRISPR-Cas arrays, as indicated by the occurrence of the core Cas proteins *cas1* and *cas2* (Makarova et al., 2011; Yosef et al., 2012; Chylinski et al., 2014), suggests that the barriers to foreign DNA acquisition may be high in *E. lacertideformus*, and may explain the absence of intact or questionable prophage regions. A single incomplete prophage region was detected, however, incomplete phages are considered cryptic or defective as they do not contain sufficient prophage genes (Arndt et al., 2016). It is therefore possible that the incomplete prophages identified were acquired prior to the acquisition of the identified CRISPR-Cas arrays, and were relevant historical promotors of genome diversity.

### Absent COGS May Explain the Inability to Grow *Enterococcus lacertideformus in vitro*

Enterococci often require rich and complex nutrients to support growth as a result of their fastidious nature (American Public Health Association, American Water Works Association, and Water Environment Federation, 2005). The abundance of COGS with metabolic properties absent from *E. lacertideformus*, encoding iron and sugar-related metabolism genes, may be correlated with its inability to grow *in vitro* (Rose et al., 2017). Iron is an essential nutrient, with its deficiency in media associated with altering or inhibiting enterococcal growth kinetics (Lisiecki, 2010). Flavodoxin/ferredoxin oxidoreductase (COG0674), encoding an enzyme demonstrating activity in enterococcal pyruvate dehydrogenation pathways, and required for growth when iron is rich or limiting (Goñi et al., 2008; Pierella Karlusich et al., 2014) was lacking in *E. lacertideformus*. Ferredoxin (COG1141) was additionally absent, and is critical for tolerance to iron starvation (Cassier-Chauvat and Chauvat, 2014). Additionally, two ferrous transport systems (Fe^2+^ transport system protein B [COG0370], Fe^2+^ transport system protein FeoA [COG1918]) were also absent, both essential for iron acquisition and in satisfying the demands for iron (Lau et al., 2016), particularly during growth (Wandersman and Delepelaire, 2004). Sugar-related metabolism genes were also lacking. Trehalose and maltose hydrolase (COG1554) did not occur in *E. lacertideformus*, and are important sources of carbon and energy to lactic acid bacteria (Creti et al., 2006). Additionally, the maltose-binding periplasmic protein (MalE) (COG2182) and ABC-type maltose transport system permease component (MalG) (COG3833) were absent and are responsible for the uptake and high affinity transport of maltodextrin and maltose across the membrane.

This study has shown that *E. lacertideformus* is missing or has lost genes associated with important metabolic pathways, including some aspects of carbohydrate and iron metabolism. As a result, *E. lacertideformus* may depend on metabolites produced by the host, or an environment generated by the host to grow, and this may explain why attempts so far to culture it using traditional bacterial isolation techniques have failed. Several approaches to culturing difficult to isolate bacteria have been recently developed (Vartoukian et al., 2010; Bodor et al., 2020). These studies suggest that co-culturing *E. lacertideformus* with other species of bacteria or in cell culture with host cells may provide required nutrients or the necessary environment for its growth. We have attempted to grow *E. lacertideformus* previously using viper fibroblasts and by inoculation of chicken eggs without success (Rose et al., 2017). However, the inoculum used had been frozen and therefore it is unknown of the bacteria in it were still viable. Therefore, other co-culturing studies using freshly obtained bacteria are indicated. Another possible reason for the failure of *E. lacertideformus* to grow in or on traditional bacterial medium is that it has evolved to use energy sources and metabolites that are only present in the host environment.

## Conclusion

**FIGURE 5:**
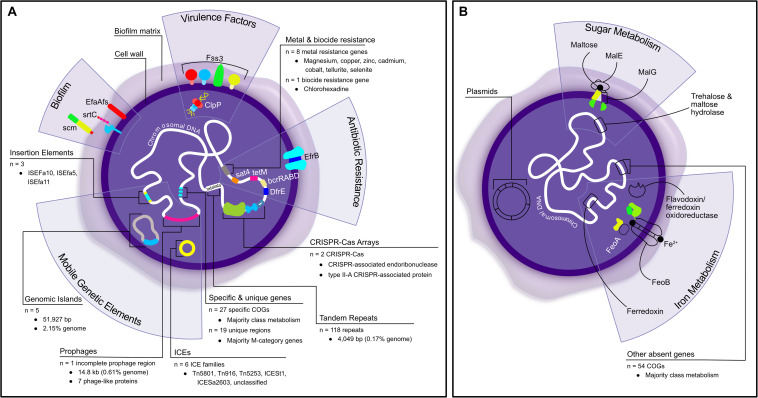
Schematic summary of the present and absent genetic elements and pathogenic properties in *E. lacertideformus* identified through *in silico* investigations into the organism’s genome. **(A)** Depicts a summary of the components identified in *E. lacertideformus*, **(B)** illustrates components identified to be lacking in the genome of *E. lacertideformus* during comparative genomics. Absent components and mechanisms are outlined with a black border only and have no color fill. Labeled descriptions for each of the components are clearly indicated.

This research has provided valuable genetic insights into the pathogenesis and evolutionary history of a novel biofilm-forming *Enterococcus* species causing mortality in Critically Endangered Christmas Island reptiles. The comprehensive genomic analysis revealed that *E. lacertideformus* may be able to adapt and respond to new environmental conditions/niches, mediated by several genetic elements it possesses (Figure 5). The identification of a relatively few antibiotic resistance genes suggests this bacterium’s pathogenicity is not human driven with limited exposure to recently developed and clinically significant antibiotics. The enhanced capacity to utilize carbohydrates and lipids, and the abundance of unique genomic loci encoding cell wall/membrane/envelop biogenesis and biofilm functionality correlates to the organism’s biofilm phenotype, which may serve to increase its pathogenicity and persistence. The inability of this bacterium to grow in traditional microbiological culture may be mediated by the absence of specific metabolism-encoding genes, likely critical for nutrient acquisition and utilization by the bacterium *in vitro* (Figure 5). The work presented here builds genomic understanding of a novel enterococcal bacterium and provides a basis for further research. Future studies related to experimental validation of the organism’s pathobiology, genotype-phenotype correlations, and elucidation of metabolic gene clusters and hypothetical protein functionality, will be required to support this genetic data, guide *in vitro* microbial cultivation, and effective therapeutic protocols for infection control and species conservation. The genes unique to *E. lacertideformus* identified in this study can further contribute to the development of a highly specific real-time PCR for disease detection in susceptible and at-risk reptiles.

## Data Availability Statement

The datasets generated for this study can be found in online repositories. The names of the repository/repositories and accession number(s) can be found in the article/Supplementary Material. Trimmed non-gecko reads from samples: 10702.133, 10706.10, and 10706.1 – Short Read Archive accession numbers: SRX9763078-SRX9763080. *Enterococcus lacertideformus* PHRS 0518 – Genbank accession number: JADAKE000000000.

## Ethics Statement

The animal study was reviewed and approved by University of Sydney Animal Ethics Committee (AEC) (2017/1211).

## Author Contributions

JA: conceptualization, investigation, methodology, software, validation, formal analysis, investigation, resources, data curation, writing – original draft preparation, writing – review and editing, visualization, project administration, and funding. DP: conceptualization, methodology, resources, writing – review and editing, supervision, project administration, and funding. KR: conceptualization, methodology, writing – review and editing, supervision, project administration, and funding. J-SE: conceptualization, software, validation, formal analysis, resources, data curation, visualization, supervision, and project administration. All authors contributed to the article and approved the submitted version.

## Conflict of Interest

The authors declare that the research was conducted in the absence of any commercial or financial relationships that could be construed as a potential conflict of interest.

## Footnotes

1 https://dfast.nig.ac.jp/

2 http://bacmet.biomedicine.gu.se/

3 https://crisprcas.i2bc.paris-saclay.fr/

4 http://crispi.genouest.org

5 https://isfinder.biotoul.fr/

6 https://phaster.ca/

7 https://antismash.secondarymetabolites.org

8 http://bagel4.molgenrug.nl/

9 http://www.pathogenomics.sfu.ca/islandviewer/

10 http://tandem.bu.edu/trf/trf.html

11 https://www.geneious.com

12 http://tree.bio.ed.ac.uk/software/figtree/

13 https://pubmlst.org/data/

## Supplementary Material

Supplementary Figure 1 16S rDNA phylogenetic tree. The evolutionary history using the 16S rDNA gene was inferred by the Maximum Likelihood method, employing the General-Time-Reversible model with Gamma distributed plus Invariant sites (GTR + G + I), with 33 nucleotide sequences including the novel *Enterococcus lacertideformus*. The percentage of trees in which the associated taxa clustered together is shown next to the branches and is derived from 1,000 bootstraps (bootstraps > 50% shown). There was a total of 1,591 positions in the final dataset.

## References

[B1] Agius J. E. Kimble B. Govendir M. Rose K. Pollard C.-L. Phalen D. N. (2020). Pharmacokinetic profile of enrofloxacin and its metabolite ciprofloxacin in Asian house geckos (Hemidactylus frenatus) after single-dose oral administration of enrofloxacin. *Vet. Anim. Sci.* 9:100116. 10.1016/j.vas.2020.100116 32734117 PMC7386737

[B2] Alcock B. P. Raphenya A. R. Lau T. T. Y. Tsang K. K. Bouchard M. Edalatmand A. (2020). CARD 2020: antibiotic resistome surveillance with the comprehensive antibiotic resistance database. *Nucleic Acids Res.* 48 D517–D525. 10.1093/nar/gkz935 31665441 PMC7145624

[B3] Altschul S. F. Gish W. Miller W. Myers E. W. Lipman D. J. (1990). Basic local alignment search tool. *J. Mol. Biol.* 215 403–410. 10.1016/S0022-2836(05)80360-2 2231712

[B4] American Public Health Association, American Water Works Association, and Water Environment Federation (2005). *Standard Methods for the Examination of Water and Wastewater.* Washington, DC: APHA-AWWA-WEF. (accessed November 10, 2020).

[B5] Andrews S. (2010). *FastQC: A Quality Control Tool for High Throughput Sequence Data.* Available online at: http://www.bioinformatics.babraham.ac.uk/projects/fastqc

[B6] Arndt D. Grant J. R. Marcu A. Sajed T. Pon A. Liang Y. (2016). PHASTER: a better, faster version of the PHAST phage search tool. *Nucleic Acids Res.* 44 W16–W21. 10.1093/nar/gkw387 27141966 PMC4987931

[B7] Ballering K. S. Kristich C. J. Grindle S. M. Oromendia A. Beattie D. T. Dunny G. M. (2009). Functional genomics of *Enterococcus faecalis*: multiple novel genetic determinants for biofilm formation in the core genome. *J. Bacteriol.* 191 2806–2814. 10.1128/jb.01688-08 19218379 PMC2668403

[B8] Bankevich A. Nurk S. Antipov D. Gurevich A. A. Dvorkin M. Kulikov A. S. (2012). SPAdes: a new genome assembly algorithm and its applications to single-cell sequencing. *J. Comput. Biol.* 19 455–477. 10.1089/cmb.2012.0021 22506599 PMC3342519

[B9] Benson G. (1999). Tandem repeats finder: a program to analyze DNA sequences. *Nucleic Acids Res.* 27 573–580. 10.1093/nar/27.2.573 9862982 PMC148217

[B10] Bertelli C. Laird M. R. Williams K. P. Simon Fraser University Research Computing Group, Lau B. Y. Hoad G. (2017). IslandViewer 4: expanded prediction of genomic islands for larger-scale datasets. *Nucleic Acids Res.* 45 W30–W35. 10.1093/nar/gkx343 28472413 PMC5570257

[B11] Beukers A. G. Zaheer R. Goji N. Amoako K. K. Chaves A. V. Ward M. P. (2017). Comparative genomics of *Enterococcus* spp. isolated from bovine feces. *BMC Microbiol.* 17:52. 10.1186/s12866-017-0962-1 28270110 PMC5341189

[B12] Blin K. Shaw S. Steinke K. Villebro R. Ziemert N. Lee S. Y. (2019). antiSMASH 5.0: updates to the secondary metabolite genome mining pipeline. *Nucleic Acids Res.* 47 W81–W87. 10.1093/nar/gkz310 31032519 PMC6602434

[B13] Bodor A. Bounedjoum N. Vincze G. E. Erdeiné Kis Á Laczi K. Bende G. (2020). Challenges of unculturable bacteria: environmental perspectives. *Rev. Environ. Sci. Bio Technol.* 19 1–22. 10.1007/s11157-020-09522-4

[B14] Bogdanov M. Heacock P. N. Dowhan W. (2002). A polytopic membrane protein displays a reversible topology dependent on membrane lipid composition. *EMBO J.* 21 2107–2116. 10.1093/emboj/21.9.2107 11980707 PMC125992

[B15] Bolger A. M. Lohse M. Usadel B. (2014). Trimmomatic: a flexible trimmer for Illumina sequence data. *Bioinformatics* 30 2114–2120. 10.1093/bioinformatics/btu170 24695404 PMC4103590

[B16] Buchfink B. Xie C. Huson D. H. (2015). Fast and sensitive protein alignment using DIAMOND. *Nat. Methods* 12 59–60. 10.1038/nmeth.3176 25402007

[B17] Bushnell B. (2014). *BBMap: A Fast, Accurate, Splice-Aware Aligner.* Available online at: https://sourceforge.net/projects/bbmap/ (accessed July 20, 2020).

[B18] Cabañes F. J. Sutton D. A. Guarro J. (2014). Chrysosporium-related fungi and reptiles: a fatal attraction. *PLoS Pathogens* 10:e1004367. 10.1371/journal.ppat.1004367 25329049 PMC4199725

[B19] Carattoli A. Zankari E. García-Fernández A. Voldby Larsen M. Lund O. Villa L. (2014). In silico detection and typing of plasmids using PlasmidFinder and plasmid multilocus sequence typing. *Antimicrob. Agents Chemother.* 58 3895–3903. 10.1128/aac.02412-14 24777092 PMC4068535

[B20] Cassier-Chauvat C. Chauvat F. (2014). Function and regulation of ferredoxins in the *Cyanobacterium*, synechocystis PCC6803: recent advances. *Life* 4 666–680. 10.3390/life4040666 25387163 PMC4284462

[B21] Chatfield C. H. Koo H. Quivey R. G. (2005). The putative autolysin regulator LytR in *Streptococcus mutans* plays a role in cell division and is growth-phase regulated. *Microbiology* 151(Pt 2) 625–631. 10.1099/mic.0.27604-0 15699211

[B22] Chen L. Zheng D. Liu B. Yang J. Jin Q. (2016). VFDB 2016: hierarchical and refined dataset for big data analysis–10 years on. *Nucleic Acids Res.* 44 D694–D697. 10.1093/nar/gkv1239 26578559 PMC4702877

[B23] Ch’ng J.-H. Chong K. K. L. Lam L. N. Wong J. J. Kline K. A. (2019). Biofilm-associated infection by enterococci. *Nat. Rev. Microbiol.* 17 82–94. 10.1038/s41579-018-0107-z 30337708 PMC13729018

[B24] Chylinski K. Makarova K. S. Charpentier E. Koonin E. V. (2014). Classification and evolution of type II CRISPR-Cas systems. *Nucleic Acids Res.* 42 6091–6105. 10.1093/nar/gku241 24728998 PMC4041416

[B25] Creti R. Koch S. Fabretti F. Baldassarri L. Huebner J. (2006). Enterococcal colonization of the gastro-intestinal tract: role of biofilm and environmental oligosaccharides. *BMC Microbiol.* 6:60. 10.1186/1471-2180-6-60 16834772 PMC1534043

[B26] Darling A. E. Mau B. Perna N. T. (2010). progressiveMauve: multiple genome alignment with gene gain, loss and rearrangement. *PLoS One* 5:e11147. 10.1371/journal.pone.0011147 20593022 PMC2892488

[B27] Feldgarden M. Brover V. Haft D. H. Prasad A. B. Slotta D. J. Tolstoy I. (2019). Validating the AMRFinder tool and resistance gene database by using antimicrobial resistance genotype-phenotype correlations in a collection of isolates. *Antimicrob. Agents Chemother.* 63:e00483-19. 10.1128/AAC.00483-19 31427293 PMC6811410

[B28] Frank K. L. Vergidis P. Brinkman C. L. Greenwood Quaintance K. E. Barnes A. M. T. Mandrekar J. N. (2015). Evaluation of the *Enterococcus faecalis* biofilm-associated virulence factors AhrC and Eep in rat foreign body osteomyelitis and in vitro biofilm-associated antimicrobial resistance. *PLoS One* 10:e0130187. 10.1371/journal.pone.0130187 26076451 PMC4467866

[B29] Furuta Y. Kawai M. Uchiyama I. Kobayashi I. (2011). Domain movement within a gene: a novel evolutionary mechanism for protein diversification. *PLoS One* 6:e18819. 10.1371/journal.pone.0018819 21533192 PMC3077401

[B30] Gan Y.-Q. Zhang T. Gan Y.-Q. Zhao Z. Zhu B. (2020). Complete genome sequences of two Enterococcus faecium strains and comparative genomic analysis. *Exp. Ther. Med.* 19 2019–2028. 10.3892/etm.2020.8447 32104261 PMC7027042

[B31] Goñi G. Serrano A. Frago S. Hervás M. Peregrina J. R. De la Rosa M. A. (2008). Flavodoxin-mediated electron transfer from photosystem I to Ferredoxin-NADP+ reductase in Anabaena:? role of flavodoxin hydrophobic residues in protein-protein interactions. *Biochemistry* 47 1207–1217. 10.1021/bi7017392 18177021

[B32] Grissa I. Vergnaud G. Pourcel C. (2007). CRISPRFinder: a web tool to identify clustered regularly interspaced short palindromic repeats. *Nucleic Acids Res.* 35 W52–W57. 10.1093/nar/gkm360 17537822 PMC1933234

[B33] Guindon S. Lethiec F. Duroux P. Gascuel O. (2005). PHYML Online–a web server for fast maximum likelihood-based phylogenetic inference. *Nucleic Acids Res.* 33 W557–W559. 10.1093/nar/gki352 15980534 PMC1160113

[B34] Gupta S. K. Padmanabhan B. R. Diene S. M. Lopez-Rojas R. Kempf M. Landraud L. (2014). ARG-ANNOT, a new bioinformatic tool to discover antibiotic resistance genes in bacterial genomes. *Antimicrob. Agents Chemother.* 58 212–220. 10.1128/aac.01310-13 24145532 PMC3910750

[B35] Gurevich A. Saveliev V. Vyahhi N. Tesler G. (2013). QUAST: quality assessment tool for genome assemblies. *Bioinformatics* 29 1072–1075. 10.1093/bioinformatics/btt086 23422339 PMC3624806

[B36] Hashem Y. A. Amin H. M. Essam T. M. Yassin A. S. Aziz R. K. (2017). Biofilm formation in enterococci: genotype-phenotype correlations and inhibition by vancomycin. *Sci. Rep.* 7:5733. 10.1038/s41598-017-05901-0 28720810 PMC5515943

[B37] Heo K. Park Y.-H. Lee K.-A. Kim J. Ham H.-I. Kim B.-G. (2019). Sugar-mediated regulation of a c-di-GMP phosphodiesterase in *Vibrio cholerae*. *Nat. Commun.* 10:5358. 10.1038/s41467-019-13353-5 31767877 PMC6877527

[B38] Holzapfel W. Wood B. (2014). *Lactic Acid Bacteria: Biodiversity And Taxonomy.* Chichester: Wiley-Blackwell.

[B39] Homan W. L. Tribe D. Poznanski S. Li M. Hogg G. Spalburg E. (2002). Multilocus sequence typing scheme for *Enterococcus faecium*. *J. Clin. Microbiol.* 40 1963–1971. 10.1128/jcm.40.6.1963-1971.2002 12037049 PMC130786

[B40] Jacobson E. R. Brown M. B. Wendland L. D. Brown D. R. Klein P. A. Christopher M. M. (2014). Mycoplasmosis and upper respiratory tract disease of tortoises: a review and update. *Vet. J.* 201 257–264. 10.1016/j.tvjl.2014.05.039 24951264

[B41] Jia B. Raphenya A. R. Alcock B. Waglechner N. Guo P. Tsang K. K. (2017). CARD 2017: expansion and model-centric curation of the comprehensive antibiotic resistance database. *Nucleic Acids Res.* 45 D566–D573. 10.1093/nar/gkw1004 27789705 PMC5210516

[B42] Juhas M. van der Meer J. R. Gaillard M. Harding R. M. Hood D. W. Crook D. W. (2009). Genomic islands: tools of bacterial horizontal gene transfer and evolution. *FEMS Microbiol. Rev.* 33 376–393. 10.1111/j.1574-6976.2008.00136.x 19178566 PMC2704930

[B43] Katoh K. Standley D. M. (2013). MAFFT multiple sequence alignment software version 7: improvements in performance and usability. *Mol. Biol. Evol.* 30 772–780. 10.1093/molbev/mst010 23329690 PMC3603318

[B44] Kawada-Matsuo M. Oogai Y. Komatsuzawa H. (2016). Sugar allocation to metabolic pathways is tightly regulated and affects the virulence of *Streptococcus mutans*. *Genes* 8:11. 10.3390/genes8010011 28036052 PMC5295006

[B45] Kumar S. Stecher G. Li M. Knyaz C. Tamura K. (2018). MEGA X: molecular evolutionary genetics analysis across computing platforms. *Mol. Biol. Evol.* 35 1547–1549. 10.1093/molbev/msy096 29722887 PMC5967553

[B46] Lau C. K. Y. Krewulak K. D. Vogel H. J. (2016). Bacterial ferrous iron transport: the Feo system. *FEMS Microbiol. Rev.* 40 273–298. 10.1093/femsre/fuv049 26684538

[B47] Lazazzera B. A. (2010). The phosphoenolpyruvate phosphotransferase system: as important for biofilm formation by *Vibrio cholerae* as it is for metabolism in *Escherichia coli*. *J. Bacteriol.* 192 4083–4085. 10.1128/JB.00641-10 20562301 PMC2916425

[B48] Lee E. W. Huda M. N. Kuroda T. Mizushima T. Tsuchiya T. (2003). EfrAB, an ABC multidrug efflux pump in *Enterococcus faecalis*. *Antimicrob. Agents Chemother.* 47 3733–3738. 10.1128/aac.47.12.3733-3738.2003 14638474 PMC296199

[B49] Li D. Liu C. M. Luo R. Sadakane K. Lam T. W. (2015). MEGAHIT: an ultra-fast single-node solution for large and complex metagenomics assembly via succinct de Bruijn graph. *Bioinformatics* 31 1674–1676. 10.1093/bioinformatics/btv033 25609793

[B50] Li H. Durbin R. (2009). Fast and accurate short read alignment with Burrows-Wheeler transform. *Bioinformatics* 25 1754–1760. 10.1093/bioinformatics/btp324 19451168 PMC2705234

[B51] Lisiecki P. (2010). [Is the iron an essential nutrient for enterococci?]. *Med. Dosw. Mikrobiol.* 62 271–280. 21114020

[B52] Liu M. Li X. Xie Y. Bi D. Sun J. Li J. (2019). ICEberg 2.0: an updated database of bacterial integrative and conjugative elements. *Nucleic Acids Res.* 47 D660–D665. 10.1093/nar/gky1123 30407568 PMC6323972

[B53] Loo C. Y. Mitrakul K. Voss I. B. Hughes C. V. Ganeshkumar N. (2003). Involvement of an inducible fructose phosphotransferase operon in *Streptococcus gordonii* biofilm formation. *J. Bacteriol.* 185 6241–6254. 10.1128/jb.185.21.6241-6254.2003 14563858 PMC219402

[B54] Lowe A. M. Lambert P. A. Smith A. W. (1995). Cloning of an Enterococcus faecalis endocarditis antigen: homology with adhesins from some oral streptococci. *Infect. Immun.* 63 703–706. 10.1128/IAI.63.2.703-706.1995 7822045 PMC173055

[B55] Mah T.-F. (2012). Biofilm-specific antibiotic resistance. *Future Microbiol.* 7 1061–1072. 10.2217/fmb.12.76 22953707

[B56] Makarova K. S. Haft D. H. Barrangou R. Brouns S. J. J. Charpentier E. Horvath P. (2011). Evolution and classification of the CRISPR-Cas systems. *Nat. Rev. Microbiol.* 9 467–477. 10.1038/nrmicro2577 21552286 PMC3380444

[B57] Martínez J. L. (2008). Antibiotics and antibiotic resistance genes in natural environments. *Science* 321 365–367. 10.1126/science.1159483 18635792

[B58] McNamara T. S. Gardiner C. Harris R. K. Hadfield T. L. Behler J. L. (1994). Streptococcal bacteremia in two Singapore house geckos (*Gekko monarchus*). *J. Zoo Wildl. Med.* 25 161–166.

[B59] Michel A. Agerer F. Hauck C. R. Herrmann M. Ullrich J. Hacker J. (2006). Global regulatory impact of ClpP protease of *Staphylococcus aureus* on regulons involved in virulence, oxidative stress response, autolysis, and DNA repair. *J. Bacteriol.* 188 5783–5796. 10.1128/JB.00074-06 16885446 PMC1540084

[B60] O’Dea M. A. Jackson B. Jackson C. Xavier P. Warren K. (2016). Discovery and partial genomic characterisation of a novel nidovirus associated with respiratory disease in wild shingleback lizards (*Tiliqua rugosa*). *PLoS One* 11:e0165209. 10.1371/journal.pone.0165209 27828982 PMC5102451

[B61] Ossiboff R. J. Caudill M. Childress A. Guzman-Vargas V. Enge K. M. Shender L. (2020). “Systemic Enterococcosis in brown anoles (Anolis sagrei) in Florida,” in *Paper presented at the Sixth Annual Wildlife and Aquatic Veterinary Disease Laboratory PALOOZA*, (Gainesville, FL).

[B62] Pal C. Bengtsson-Palme J. Rensing C. Kristiansson E. Larsson D. G. J. (2014). BacMet: antibacterial biocide and metal resistance genes database. *Nucleic Acids Res.* 42 D737–D743. 10.1093/nar/gkt1252 24304895 PMC3965030

[B63] Peng Z. Li M. Wang W. Liu H. Fanning S. Hu Y. (2017). Genomic insights into the pathogenicity and environmental adaptability of *Enterococcus hirae* R17 isolated from pork offered for retail sale. *MicrobiologyOpen* 6:e00514. 10.1002/mbo3.514 28799224 PMC5727370

[B64] Pierella Karlusich J. J. Lodeyro A. F. Carrillo N. (2014). The long goodbye: the rise and fall of flavodoxin during plant evolution. *J. Exp. Bot.* 65 5161–5178. 10.1093/jxb/eru273 25009172 PMC4400536

[B65] Pillai S. K. Sakoulas G. Eliopoulos G. M. Moellering R. C. Jr. Murray B. E. Inouye R. T. (2004). Effects of glucose on fsr-mediated biofilm formation in *Enterococcus faecalis*. *J. Infect Dis.* 190 967–970. 10.1086/423139 15295702

[B66] Rissman A. I. Mau B. Biehl B. S. Darling A. E. Glasner J. D. Perna N. T. (2009). Reordering contigs of draft genomes using the Mauve aligner. *Bioinformatics* 25 2071–2073. 10.1093/bioinformatics/btp356 19515959 PMC2723005

[B67] Rodrigues M. V. Borges N. Santos H. (2016). Glycerol phosphate Cytidylyltransferase stereospecificity is key to understanding the distinct stereochemical compositions of Glycerophosphoinositol in bacteria and archaea. *Appl. Environ. Microbiol.* 83:e02462-16. 10.1128/AEM.02462-16 27795311 PMC5165115

[B68] Rose K. Agius J. Hall J. Thompson P. Eden J.-S. Srivastava M. (2017). Emergent multisystemic *Enterococcus* infection threatens endangered Christmas Island reptile populations. *PLoS One* 12:e0181240. 10.1371/journal.pone.0181240 28727845 PMC5519069

[B69] Rousseau C. Gonnet M. Le Romancer M. Nicolas J. (2009). CRISPI: a CRISPR interactive database. *Bioinformatics* 25 3317–3318. 10.1093/bioinformatics/btp586 19846435 PMC2788928

[B70] Ruiz-Garbajosa P. Bonten M. J. M. Robinson D. A. Top J. Nallapareddy S. R. Torres C. (2006). Multilocus sequence typing scheme for *Enterococcus faecalis* reveals hospital-adapted genetic complexes in a background of high rates of recombination. *J. Clin. Microbiol.* 44 2220–2228. 10.1128/JCM.02596-05 16757624 PMC1489431

[B71] Salvadori M. Vito K. M. (2015). Pharmacokinetics of Enrofloxacin and its metabolite ciprofloxacin after intracoelomic administration in tortoises (*Testudo hermanni*). *Israel J. Vet. Med.* 70 45–48.

[B72] Schroder J. (1989). Enrofloxacin: a new antimicrobial agent. *J. S. Afr. Vet. Assoc.* 60 122–124. 2691696

[B73] Seemann T. (2020). *ABRicate.* Available online at: https://github.com/tseemann/abricate (accessed May 5, 2020).

[B74] Siguier P. Perochon J. Lestrade L. Mahillon J. Chandler M. (2006). ISfinder: the reference centre for bacterial insertion sequences. *Nucleic Acids Res.* 34 D32–D36. 10.1093/nar/gkj014 16381877 PMC1347377

[B75] Sillanpää J. Chang C. Singh K. V. Montealegre M. C. Nallapareddy S. R. Harvey B. R. (2013). Contribution of individual Ebp Pilus subunits of *Enterococcus faecalis* OG1RF to pilus biogenesis, biofilm formation and urinary tract infection. *PLoS One* 8:e68813. 10.1371/journal.pone.0068813 23874774 PMC3708956

[B76] Sillanpää J. Nallapareddy S. R. Houston J. Ganesh V. K. Bourgogne A. Singh K. V. (2009). A family of fibrinogen-binding MSCRAMMs from *Enterococcus faecalis*. *Microbiology (Reading, England)* 155(Pt 7) 2390–2400. 10.1099/mic.0.027821-0 19389755 PMC2739004

[B77] Sillanpää J. Nallapareddy S. R. Prakash V. P. Qin X. Höök M. Weinstock G. M. (2008). Identification and phenotypic characterization of a second collagen adhesin, Scm, and genome-based identification and analysis of 13 other predicted MSCRAMMs, including four distinct pilus loci, in *Enterococcus faecium*. *Microbiology (Reading, England)* 154(Pt 10) 3199–3211. 10.1099/mic.0.2008/017319-0 18832325 PMC2677164

[B78] Singh K. V. Coque T. M. Weinstock G. M. Murray B. E. (1998). In vivo testing of an *Enterococcus faecalis* efaA mutant and use of efaA homologs for species identification. *FEMS Immunol. Med. Microbiol.* 21 323–331. 10.1111/j.1574-695X.1998.tb01180.x 9753005

[B79] Stothard P. Grant J. R. Van Domselaar G. (2019). Visualizing and comparing circular genomes using the CGView family of tools. *Brief. Bioinform.* 20 1576–1582. 10.1093/bib/bbx081 28968859 PMC6781573

[B80] Tanizawa Y. Fujisawa T. Nakamura Y. (2018). DFAST: a flexible prokaryotic genome annotation pipeline for faster genome publication. *Bioinformatics* 34 1037–1039. 10.1093/bioinformatics/btx713 29106469 PMC5860143

[B81] Tetzlaff S. J. Ravesi M. J. Allender M. C. Carter E. T. Degregorio B. A. Josimovich J. M. (2017). Snake fungal disease affects behavior of free-ranging massasauga rattlesnakes (*Sistrurus catenatus*). *Herpetol. Conserv. Biol.* 12 624–634.

[B82] Theilacker C. Sanchez-Carballo P. Toma I. Fabretti F. Sava I. Kropec A. (2009). Glycolipids are involved in biofilm accumulation and prolonged bacteraemia in *Enterococcus faecalis*. *Mol. Microbiol.* 71 1055–1069. 10.1111/j.1365-2958.2008.06587.x 19170884

[B83] van Heel A. J. de Jong A. Song C. Viel J. H. Kok J. Kuipers O. P. (2018). BAGEL4: a user-friendly web server to thoroughly mine RiPPs and bacteriocins. *Nucleic Acids Res.* 46 W278–W281. 10.1093/nar/gky383 29788290 PMC6030817

[B84] Vartoukian S. R. Palmer R. M. Wade W. G. (2010). Strategies for culture of ‘unculturable’ bacteria. *FEMS Microbiol. Lett.* 309 1–7. 10.1111/j.1574-6968.2010.02000.x 20487025

[B85] Vasu K. Nagaraja V. (2013). Diverse functions of restriction-modification systems in addition to cellular defense. *Microbiol. Mol. Biol. Rev.* 77:53. 10.1128/MMBR.00044-12 23471617 PMC3591985

[B86] Wandersman C. Delepelaire P. (2004). Bacterial iron sources: from siderophores to hemophores. *Annu. Rev. Microbiol.* 58 611–647. 10.1146/annurev.micro.58.030603.123811 15487950

[B87] Willett J. L. E. Ji M. M. Dunny G. M. (2019). Exploiting biofilm phenotypes for functional characterization of hypothetical genes in *Enterococcus faecalis*. *NPJ Biofilms Microbiomes* 5:23. 10.1038/s41522-019-0099-0 31552139 PMC6753144

[B88] Xiao R. (2019). *POEM py3k: GitHub repository.* Available online at: https://github.com/Rinoahu/POEM_py3k (accessed December 16, 2020).

[B89] Yang B. Lei Z. Zhao Y. Ahmed S. Wang C. Zhang S. (2017). Combination susceptibility testing of common antimicrobials in vitro and the effects of Sub-MIC of antimicrobials on *Staphylococcus aureus* biofilm formation. *Front. Microbiol.* 8:2125. 10.3389/fmicb.2017.02125 29163415 PMC5671985

[B90] Yosef I. Goren M. G. Qimron U. (2012). Proteins and DNA elements essential for the CRISPR adaptation process in *Escherichia coli*. *Nucleic Acids Res.* 40 5569–5576. 10.1093/nar/gks216 22402487 PMC3384332

[B91] Zankari E. Allesøe R. Joensen K. G. Cavaco L. M. Lund O. Aarestrup F. M. (2017). PointFinder: a novel web tool for WGS-based detection of antimicrobial resistance associated with chromosomal point mutations in bacterial pathogens. *J. Antimicrob. Chemother.* 72 2764–2768. 10.1093/jac/dkx217 29091202 PMC5890747

[B92] Zankari E. Hasman H. Cosentino S. Vestergaard M. Rasmussen S. Lund O. (2012). Identification of acquired antimicrobial resistance genes. *J. Antimicrob. Chemother.* 67 2640–2644. 10.1093/jac/dks261 22782487 PMC3468078

[B93] Zhong Z. Zhang W. Song Y. Liu W. Xu H. Xi X. (2017). Comparative genomic analysis of the genus Enterococcus. *Microbiol. Res.* 196 95–105. 10.1016/j.micres.2016.12.009 28164795

[B94] Zwart P. Cornelisse J. (1972). “Streptockokensepsis mit Hautwucherungen bei Eidechsen,” in *Paper presented at the Verhandlungsbericht des XIV Internationalen Symposiums über die Erkrankungen der Zootiere*, (Wrocław).

