# Supplementary figures and images for: Genomic Insights Into the Pathogenicity of a Novel Biofilm-Forming Enterococcus sp. Bacteria (Enterococcus lacertideformus) Identified in Reptiles
Source: Front Microbiol. 2021 Mar 2;12:635208. doi: 10.3389/fmicb.2021.635208 (PMC7960928; doi:10.3389/fmicb.2021.635208)

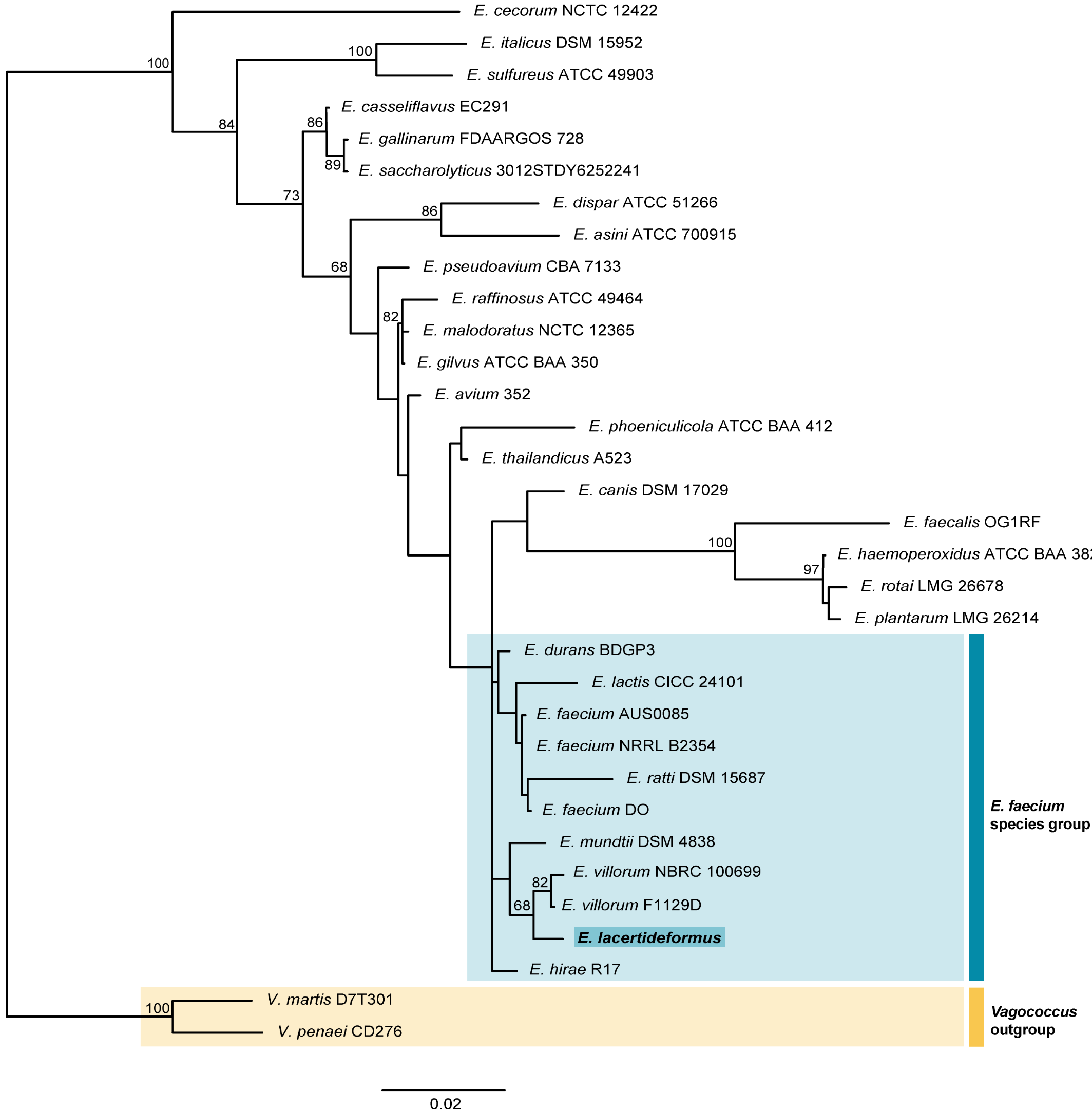

Supplement: Supplementary Figure 1 — 16S rDNA phylogenetic tree. The evolutionary history using the 16S rDNA gene was inferred by the Maximum Likelihood method, employing the General-Time-Reversible model with Gamma distributed plus Invariant sites (GTR + G + I), with 33 nucleotide sequences including the novel Enterococcus lacertideformus. The percentage of trees in which the associated taxa clustered together is shown next to the branches and is derived from 1,000 bootstraps (bootstraps > 50% shown). There was a total of 1,591 positions in the final dataset. [file Image_1.TIFF]
