# Supplementary material for: Genomic Insights Into the Pathogenicity of a Novel Biofilm-Forming Enterococcus sp. Bacteria (Enterococcus lacertideformus) Identified in Reptiles
Source: Front Microbiol. 2021 Mar 2;12:635208. doi: 10.3389/fmicb.2021.635208 (PMC7960928; doi:10.3389/fmicb.2021.635208)
Supplement: Supplementary file 2 [file Data_Sheet_1.docx]

**Supplementary Data**

**Supplementary Table 1:** List of ordered contigs of the *E. lacertideformus* whole genome shotgun assembly.

| **Contig** | **Start position** | **End position** | **Length of contig** | **Contig** | **Start position** | **End position** | **Length of contig** |
| --- | --- | --- | --- | --- | --- | --- | --- |
| **1** | 1 | 43,878 | 43,878 | **21** | 2,106,758 | 2,128,317 | 21,560 |
| **2** | 43,879 | 137,978 | 94,100 | **22** | 2,128,318 | 2,255,368 | 127,051 |
| **3** | 137,979 | 216,275 | 78,297 | **23** | 2,255,369 | 2,392,631 | 137,263 |
| **4** | 216,276 | 273,522 | 57,247 | **24** | 2,392,632 | 2,408,233 | 15,062 |
| **5** | 273,523 | 398,178 | 124,656 | **25** | 2,408,234 | 2,409,773 | 1,540 |
| **6** | 398,179 | 410,122 | 11,944 | **26** | 2,409,774 | 2,414,696 | 4,923 |
| **7** | 410,123 | 465,698 | 55,576 | **27** | 2,414,697 | 2,414,965 | 269 |
| **8** | 465,699 | 628,880 | 162,182 | **28** | 2,414,966 | 2,415,235 | 270 |
| **9** | 628,881 | 674,564 | 45,684 | **29** | 2,415,236 | 2,416,214 | 979 |
| **10** | 674,565 | 855,267 | 180,703 | **30** | 2,416,215 | 2,416,603 | 389 |
| **11** | 855,268 | 856,355 | 1,088 | **31** | 2,416,604 | 2,417,172 | 569 |
| **12** | 856,356 | 860,735 | 4,380 | **32** | 2,417,173 | 2,417,508 | 336 |
| **13** | 860,736 | 975,120 | 114,385 | **33** | 2,417,509 | 2,417,850 | 342 |
| **14** | 975,121 | 1,107,858 | 132,738 | **34** | 2,417,851 | 2,418,127 | 277 |
| **15** | 1,107,859 | 1,196,502 | 88,644 | **35** | 2,418,128 | 2,418,400 | 273 |
| **16** | 1,196,503 | 1,627,765 | 431,263 | **36** | 2,418,401 | 2,418,788 | 388 |
| **17** | 1,627,766 | 1,905,691 | 277,926 | **37** | 2,418,789 | 2,419,057 | 269 |
| **18** | 1,905,692 | 1,939,205 | 33,514 | **38** | 2,419,058 | 2,419,406 | 349 |
| **19** | 1,939,206 | 1,958,302 | 19,097 | **39** | 2,419,407 | 2,419,934 | 528 |
| **20** | 1,958,303 | 2,106,757 | 148,455 |  |  |  |  |

**Supplementary Table 2:** Integrative conjugative elements identified in *E. lacertideformus*.

| **ICEberg ID** | **ICE name** | **ICE family** | **Organism carrying ICE** | **Accession ID** | **Genome coordinates** | **Insertion site** | **Function** | **Score (bits)** | **E-value** | **ID (%)** |
| --- | --- | --- | --- | --- | --- | --- | --- | --- | --- | --- |
| 224 | Tn5801 | Tn5801 | *Staphylococcus aureus* subsp. *aureus* Mu50 | BA000017 | Contig 17  (1674444-1677195) | GMP-synthase gene (SAV0391) | Tetracycline resistance | 868.9 | 0 | 68 |
| 424 | ICESauMu3-1 | Tn5801 | *Staphylococcus aureus* subsp. *aureus* Mu3 | AP009324 | Contig 17  (1674444-1677195) |  |  | 868.9 | 0 | 68 |
| 428 | ICESauJKD6008-2 | Tn5801 | *Staphylococcus aureus* subsp. *aureus* str. JKD6008 | CP002120 | Contig 17  (1674444-1677195) |  |  | 867.6 | 0 | 68 |
| 429 | ICESauT0131-2 | Tn5801 | *Staphylococcus aureus* subsp. *aureus* T0131 | CP002643 | Contig 17  (1674444-1677195) |  | - | 864.9 | 0 | 68 |
| 430 | ICESauTW20-2 | Tn5801 | *Staphylococcus aureus* subsp. *aureus* TW20 | FN433596 | Contig 17  (1674444-1677195) |  |  | 726.8 | 0 | 65 |
| 289 | ICELm1 | Tn5801 | *Listeria monocytogenes* EGD-e | AL591824 | Contig 17  1675814-1677434 | 3' End of a gene encoding a GMP synthase | Putative cadmium resistance | 504.9 | 0 | 67 |
| 323 | ICECdiM120-1 | Tn916 | *Clostridium difficile* M120 | FN665653 | Contig 17  1675813-1677195 | AT rich regions | - | 491.9 | 0 | 70 |
| 232 | ICESsu(BM407)1 | Unclassified | *Streptococcus suis* BM407 | FM252032 | Contig 17  1675813-1677195 | luciferase-like monooxygenase | Tetracycline resistance | 491.7 | 0 | 70 |
| 234 | ICESsu(SC84) | ICESa2603 | *Streptococcus suis* SC84 | FM252031 | Contig 17  1675813-1677195 | The 3' end of 50S rDNA | Tetracycline resistance | 490.4 | 0 | 70 |
| 84 | ICESpn11930 | Tn5253 | *Streptococcus pneumoniae* 11930 | FR671403 | Contig 17  1675813-1677195 |  | Tetracycline resistance | 490.4 | 0 | 70 |
| 364 | Tn5253 | Tn5253 | *Streptococcus* *pneumoniae* strain DP1322 | EU351020 | Contig 17  1675813-1677195 |  | - | 490.4 | 0 | 70 |
| 829 | ICESsu05SC260 | Unclassified | *Streptococcus* *suis* 05SC260 | KX077888 | Contig 17  1675813-1677195 | rplL | Antibiotic resistance genes: sat4, tet(M) | 490.4 | 0 | 70 |
| 88 | ICESpn11876 | Tn5253 | *Streptococcus* *pneumoniae* 11876 | FR671404 | Contig 17  1675813-1677195 |  | - | 490.4 | 0 | 70 |
| 821 | ICESpnDCC1902 | Tn5253 | *Streptococcus* *pneumoniae* DCC1902 | HG799491 | Contig 17  1675813-1677195 |  | - | 490.4 | 0 | 70 |
| 820 | ICESpnDCC1738 | Tn5253 | *Streptococcus* *pneumoniae* DCC1738 | HG799492 | Contig 17  1675813-1677195 |  | - | 490.4 | 0 | 70 |
| 815 | ICESpn22664 | Tn5253 | *Streptococcus* *pneumoniae* 22664 | HG799489 | Contig 17  1675813-1677195 |  | - | 490.4 | 0 | 70 |
| 372 | ICESpnP1031-1 | Tn5253 | *Streptococcus* *pneumoniae* P1031 | CP000920 | Contig 17  1675813-1677195 |  | Tetracycline resistance | 490.4 | 0 | 70 |
| 373 | ICESpn6706B-1 | Tn5253 | *Streptococcus* *pneumoniae* 670-6B | CP002176 | Contig 17  1675813-1677195 | GTP-binding protein gene | Tetracycline resistance | 490.4 | 0 | 70 |
| 322 | ICESgal1 | Unclassified | *Streptococcus* *gallolyticus* UCN34 | FN597254 | Contig 17  1675813-1677195 |  | Tetracycline resistance | 490.4 | 0 | 70 |
| 306 | Tn925 | Tn916 | *Enterococcus* *faecalis* plasmid pCF10 | AY855841 | Contig 17  1675813-1677195 | AT rich regions | - | 490.4 | 0 | 70 |
| 304 | ICESga43143-1 | Tn916 | *Streptococcus* *gallolyticus* subsp. *gallolyticus* ATCC 43143 | AP012053 | Contig 17  1675813-1677195 | AT rich regions | Tetracycline resistance | 490.4 | 0 | 70 |
| 311 | ICESsu05ZYH33-1 | ICESa2603 | *Streptococcus* *suis* 05ZYH33 | CP000407 | Contig 17  1675813-1677195 | AT rich regions |  | 490.4 | 0 | 70 |
| 310 | ICESpsHKU1003-1 | Tn916 | *Staphylococcus* *pseudintermedius* HKU10-03 | CP002439 | Contig 17  1675813-1677195 | AT rich regions | Tetracycline resistance | 490.4 | 0 | 70 |
| 305 | ICESauST398-1 | Tn916 | *Staphylococcus* *aureus* subsp. *aureus* ST398 | AM990992 | Contig 17  1675813-1677195 | AT rich regions | Tetracycline resistance | 490.4 | 0 | 70 |
| 309 | ICESag2603VR-1 | Tn916 | *Streptococcus* *agalactiae* 2603V/R | AE009948 | Contig 17  1675813-1677195 | AT rich regions | Tetracycline resistance | 490.4 | 0 | 70 |
| 313 | ICESsu98HAH33-1 | ICESa2603 | *Streptococcus* *suis* 98HAH33 | CP000408 | Contig 17  1675813-1677195 | AT rich regions | - | 490.4 | 0 | 70 |
| 47 | Tn5251 | Tn916 | *Streptococcus* *pneumoniae* DP1322 | FJ711160 | Contig 17  1675813-1677195 | AT rich regions | Tetracycline resistance | 490.4 | 0 | 70 |
| 331 | ICESpnH19A6-1 | Tn916 | *Streptococcus* *pneumoniae* Hungary 19A-6 | CP000936 | Contig 17  1675813-1677195 | AT rich regions | Tetracycline resistance | 489.0 | 0 | 70 |
| 402 | CTn6 | Tn916 | *Clostridium* *difficile* 630 | AM180355 | Contig 17  1675816-1677236 |  | - | 484.1 | 0 | 69 |
| 403 | CTn7 | Tn916 | *Clostridium* *difficile* 630 | AM180355 | Contig 17  1675811-1677195 |  | - | 482.7 | 0 | 70 |
| 398 | CTn1 | Tn916 | *Clostridium* *difficile* 630 | AM180355 | Contig 17  1675811-1677269 |  | - | 480.8 | 0 | 69 |
| 327 | ICEFal35896-1 | Tn916 | *Filifactor* *alocis* ATCC 35896 | CP002390 | Contig 17  1675813-1677201 | AT rich regions | Tetracycline resistance | 470.6 | 0 | 69 |
| 770 | ICECp1 | Tn916 | *Clostridium* *perfringens* JIR12708 | LN835295 | Contig 17  1675813-1677197 |  | Bacitracin resistance determinant (bcrRABD) | 449.4 | 0 | 38 |
| 78 | ICESt1 | ICESt1 | *Streptococcus* *thermophilus* CNRZ368 | AJ278471 | Contig 22  2152064-2153659 | fda | Type II Restriction-Modification System | 718.1 | 1.3e-202 | 76 |
| 76 | ICESde3396 | ICESa2603 | *Streptococcus* *dysgalactiae* subsp. *equisimilis* NS3396 | EU142041 | Contig 23  2339177-2340565 |  | Cadmium resistance; arsenic resistance | 510.2 | 3.6e-179 | 70 |
| 792 | ICESag37741 | Unclassified | *Streptococcus* *agalactiae* CCUG 37741 | ALQQ01000033 | Contig 23  2339177-2340565 |  | - | 508.8 | 9.6e-179 | 70 |

**Supplementary Table 3:** Prophage proteins identified in the *E. lacertideformus* assembly.

| **Prophage hit** | **Product** | **Phage family** | **Position** | **E-value** | **NCBI accession** |
| --- | --- | --- | --- | --- | --- |
| Gordonia phage Ghobes | DNA polymerase | Siphoviridae | Contig 15 1125949-1127256 | 1.27e-20 | NC031028 |
| Prochlorococcus phage Syn1 | Phosphoribosylaminoimidazole-succinocarboxamide synthase | Myoviridae | Contig 15 1128346-1129062 | 2.10e-59 | NC015288 |
| Microbacterium phage Min1 | Putative phosphoribosyl formylglycinamidine (FGAM) synthase II | Siphoviridae | Contig 15 1130095-1131321  Contig 15 1131331-1132230 | 2.23e-114  3.59e-54 | NC009603 |
| Synechococcus phage ACG-2014i isolate Syn7803US120 | Phosphoribosylaminoimidazole synthetase | Myoviridae | Contig 15 1133672-1134715 | 1.31e-75 | NC027132 |
| Prochlorococcus phage P-SSM7 | PurH2 | Myoviridae | Contig 15 1135298-1135648 | 5.74e-38 | NC015290 |
| Prochlorococcus phage P-SSM2 | 5-aminoimidazole-4-carboxamide ribonucleotide formyltransferase/IMP cyclohydrolase | Myoviridae | Contig 15 1135911-1136819 | 1.48e-90 | NC006883 |
| Bacillus phage G | Gp120 | Myoviridae | Contig 15 1139799-1140776 | 0.0 | NC023719 |

**Supplementary Table 4:** Insertion elements identified in *E. lacertideformus*.

| **IS element** | **IS family** | **Group** | **IS length** | **IR length (bp)** | **Transposition** | **Hit size bp** | **Genome coordinates** | **Origin** | **E-value** | **NCBI accession** |
| --- | --- | --- | --- | --- | --- | --- | --- | --- | --- | --- |
| ISEfa10 | IS3 | IS50 | 1561 | 22/24 | ND | 657 | Contig 13  873155-873810 | *E. faecium* C1706 | 0.0 | FJ795373 |
| ISEfa5 | ISL3 |  | 1514 | 16/25 | Y | 208 | Contig 2  103002-103209 | *E. faecium* | 2e-66 | AY495588 |
| ISEfa11 | ISL3 |  | 1514 | 17/26 | ND | 308 | Contig 2  103002-103302 | *E. faecium* C864 | 6e-64 | FJ866609 |

IR, inverted repeats; IS, insertion; ND, not determined; Y, yes.

**Supplementary Table 5:** Genomic islands identified in *E. lacertideformus*.

| **Locus** | **Prediction Method** | **Start** | **Stop** | **Length (bp)** | **Strand** | **Gene name** | **Gene product name** |
| --- | --- | --- | --- | --- | --- | --- | --- |
| **GI 1 (Contig 2)** | **IslandPath-DIMOB** | **76041** | **96229** | **7608** |  |  |  |
| 00760 |  | 75854 | 76138 | 284 | - |  | *Recombinase family protein |
| 00770 |  | 76041 | 76313 | 272 | - |  | Hypothetical protein |
| 00780 |  | 76564 | 77247 | 683 | - | rpiA | Ribose-5-phosphate isomerase A |
| 00790 |  | 77556 | 77969 | 413 | + | rpsL | 30S ribosomal protein S12 |
| 00800 |  | 78057 | 78527 | 470 | + | rpsG | 30S ribosomal protein S7 |
| 00810 |  | 78606 | 80690 | 2084 | + | fusA | Elongation factor G |
| 00820 |  | 80862 | 82049 | 1187 | + | tufA | Elongation factor Tu |
| 00830 |  | 82532 | 82840 | 308 | + |  | *30S ribosomal protein S10 |
| 00840 |  | 82872 | 83540 | 668 | + | rplC | 50S ribosomal protein L3 |
| 00850 |  | 83528 | 84151 | 623 | + | rplD | 50S ribosomal protein L4 |
| 00860 |  | 84151 | 84441 | 290 | + | rplW | 50S ribosomal protein L23 |
| 00870 |  | 84481 | 85314 | 833 | + | rplB | 50S ribosomal protein L2 |
| 00880 |  | 85357 | 85635 | 278 | + | rpsS | 30S ribosomal protein S19 |
| 00890 |  | 85657 | 86004 | 347 | + | rplV | 50S ribosomal protein L22 |
| 00900 |  | 86018 | 86674 | 656 | + | rpsC | 30S ribosomal protein S3 |
| 00910 |  | 86677 | 87111 | 434 | + | rplP | 50S ribosomal protein L16 |
| 00920 |  | 87101 | 87289 | 188 | + | rpmC | 50S ribosomal protein L29 |
| 00930 |  | 87314 | 87580 | 266 | + | rpsQ | 30S ribosomal protein S17 |
| 00940 |  | 87638 | 88006 | 368 | + | rplN | 50S ribosomal protein L14 |
| 00950 |  | 88040 | 88342 | 302 | + | rplX | 50S ribosomal protein L24 |
| 00960 |  | 88379 | 88918 | 539 | + | rplE | 50S ribosomal protein L5 |
| 00970 |  | 88937 | 89122 | 185 | + | rpsZ | 30S ribosomal protein S14 type Z |
| 00980 |  | 89159 | 89557 | 398 | + | rpsH | 30S ribosomal protein S8 |
| 00990 |  | 89589 | 90125 | 536 | + | rplF | 50S ribosomal protein L6 |
| 01000 |  | 90285 | 90641 | 356 | + | rplR | 50S ribosomal protein L18 |
| 01010 |  | 90662 | 91162 | 500 | + | rpsE | 30S ribosomal protein S5 |
| 01020 |  | 91177 | 91356 | 179 | + | rpmD | 50S ribosomal protein L30 |
| 01030 |  | 91410 | 91850 | 440 | + | rplO | 50S ribosomal protein L15 |
| 01040 |  | 91850 | 93145 | 1295 | + | secY | Protein translocase subunit SecY |
| 01050 |  | 93205 | 93852 | 647 | + | adk | Adenylate kinase |
| 01060 |  | 94044 | 94262 | 218 | + | infA | Translation initiation factor IF-1 |
| 01070 |  | 94429 | 94794 | 365 | + | rpsM | 30S ribosomal protein S13 |
| 01080 |  | 94822 | 95211 | 389 | + | rpsK | 30S ribosomal protein S11 |
| 01090 |  | 95291 | 96229 | 938 | + | rpoA | DNA-directed RNA polymerase subunit alpha |
| **GI 2 (Contig 3)** | **SIGI-HMM** | **167048** | **176306** | **6258** |  |  |  |
| 01870 |  | 166704 | 167051 | 347 | + |  | Hypothetical protein |
| 01880 |  | 167048 | 167317 | 269 | + |  | Hypothetical protein |
| 01890 |  | 167336 | 168250 | 914 | + | LytR2 | Transcriptional regulator LytR |
| 01900 |  | 168291 | 168650 | 359 | + |  | Hypothetical protein |
| 01910 |  | 170109 | 171026 | 917 | - |  | *Helix-turn-helix domain-containing protein |
| 01920 |  | 172172 | 172672 | 500 | + |  | Hypothetical protein |
| 01930 |  | 172783 | 173388 | 605 | + |  | Hypothetical protein |
| 01940 |  | 173570 | 174373 | 803 | + |  | *O-antigen ligase family protein |
| 01950 |  | 174318 | 174527 | 209 | - |  | Hypothetical protein |
| 01960 |  | 174644 | 174778 | 134 | + |  | Hypothetical protein |
| 01970 |  | 175103 | 175765 | 662 | + |  | *Glycosyltransferase family 4 protein |
| 01980 |  | 175767 | 176306 | 539 | + |  | *Acyltransferase |
| **GI 3 (Contig 14)** | **IslandPath-DIMOB** | **1057333** | **1062337** | **3826** |  |  |  |
| 12140 |  | 1057333 | 1058028 | 695 | + |  | *DegV family protein |
| 12150 |  | 1058066 | 1058236 | 170 | - |  | Hypothetical protein |
| 12160 |  | 1058261 | 1058797 | 536 | - |  | *ISL3 family transposase |
| 12170 |  | 1059300 | 1060520 | 1220 | + |  | Hypothetical protein |
| 12180 |  | 1060872 | 1061111 | 239 | + |  | Hypothetical protein |
| 12190 |  | 1061077 | 1061583 | 506 | + |  | Hypothetical protein |
| 12200 |  | 1061794 | 1062027 | 233 | + |  | Hypothetical protein |
| 12210 |  | 1062110 | 1062337 | 227 | + |  | Hypothetical protein |
| **GI 4 (Contig 16)** | **IslandPath-DIMOB** | **1542902** | **1547266** | **3031** |  |  |  |
| 17660 |  | 1542902 | 1543330 | 428 | - |  | *Site-specific recombinase, phage integrase family |
| 17670 |  | 1543351 | 1543866 | 515 | - |  | *tyrosine-type recombinase/integrase |
| 17680 |  | 1544055 | 1544282 | 227 | - |  | Hypothetical protein |
| 17690 |  | 1544322 | 1544462 | 140 | - |  | Hypothetical protein |
| 17700 |  | 1545232 | 1545621 | 389 | + |  | *Response regulator |
| 17710 |  | 1545618 | 1545839 | 221 | + |  | Hypothetical protein |
| 17720 |  | 1546084 | 1546563 | 479 | - |  | *GHKL domain-containing protein |
| 17730 |  | 1546634 | 1547266 | 632 | - |  | *GHKL domain-containing protein |
| **GI 5 (Contig 22)** | **IslandPath-DIMOB** | **2142236** | **2155349** | **8475** |  |  |  |
| 24340 |  | 2142236 | 2142385 | 149 | - |  | Hypothetical protein |
| 24350 |  | 2143268 | 2145502 | 2234 | - |  | *FAD-dependant oxidoreductase |
| 24360 |  | 2147241 | 2147384 | 143 | + |  | Hypothetical protein |
| 24370 |  | 2147610 | 2148254 | 644 | + |  | *M28 family peptidase |
| 24380 |  | 2149014 | 2150231 | 1217 | - |  | Methyltransferase |
| 24390 |  | 2150221 | 2150877 | 656 | - |  | Restriction endonuclease |
| 24400 |  | 2151065 | 2151805 | 740 | - |  | *Recombinase family protein |
| 24410 |  | 2151827 | 2152030 | 203 | - |  | *Recombinase family protein |
| 24420 |  | 2152002 | 2152841 | 839 | - |  | Hypothetical protein |
| 24430 |  | 2152842 | 2152994 | 152 | - |  | Hypothetical protein |
| 24440 |  | 2153033 | 2153215 | 182 | - |  | Hypothetical protein |
| 24450 |  | 2153237 | 2153572 | 335 | - |  | Hypothetical protein |
| 24460 |  | 2153780 | 2154037 | 257 | - |  | Hypothetical protein |
| 24470 |  | 2154044 | 2154514 | 470 | - |  | Hypothetical protein |
| 24480 |  | 2154547 | 2154801 | 254 | - |  | Hypothetical protein |
| 24490 |  | 2154981 | 2155349 | 368 | - |  | *23S rRNA |

GI, genomic island; *, Genes that produced significant BLASTp results following initial characterisation as a hypothetical protein with Islandviewer 4.

**Supplementary Table 6:** BLASTp data and functions of hypothetical proteins identified using the Islandviewer program.

| **Genomic Island** | **Locus** | **Gene product name** | **Organism** | **E-value** | **Identity (%)** | **Query (%)** | **NCBI accession** | **Function** |
| --- | --- | --- | --- | --- | --- | --- | --- | --- |
| GI-1 | 00760 | Recombinase family protein | *Enterococcus hirae* | 2e-52 | 97.0 | 92.4 | WP161652162.1 | Site-specific recombination of DNA molecules by a concerted, four-strand cleavage and rejoining mechanism. |
| GI-2 | 01910 | Helix-turn-helix domain-containing protein | *Enterococcus hirae* | 7e-60 | 97.0 | 39.5 | WP179287063.1 | DNA-binding protein that activates the expression of several important virulence genes in response to changing environmental conditions |
| GI-2 | 01940 | O-antigen ligase family protein | *Enterococcus hirae* | 1e-73 | 93.0 | 49.8 | WP095454090.1 | Synthesis of O-antigen, a lipopolysaccharide found in the outer membrane of bacteria. |
| GI-2 | 01970 | Glycosyltransferase family 4 protein | *Enterococcus hirae* | 1e-95 | 96.0 | 64.6 | WP143716033.1 | Catalyse the transfer of sugar moieties from activated donor molecules to specific acceptor molecules, forming glycosidic bonds. |
| GI-2 | 01980 | Acyltransferase | *Enterococcus hirae* | 2e-86 | 97.0 | 70.3 | WP053766520.1 | Biosynthesis of colanic acid, an exopolysaccharide expressed in some bacteria. |
| GI-3 | 12140 | DegV family protein | *Enterococcus villorum* | 9e-156 | 100.0 | 91.3 | WP081185133.1 | Binding of fatty-acids and may play a role in the cellular functions of fatty acid transport or metabolism. |
| GI-3 | 12160 | ISL3 family transposase | *Enterococcus durans* | 1e-109 | 98.0 | 88.1 | WP113846169.1 | Efficient DNA transposition. |
| GI-4 | 17660 | Site-specific recombinase, phage integrase family | *Enterococcus faecalis* | 2e-88 | 100.0 | 87.3 | EPH96785.1 | DNA integration and recombination, and viral genome integration into host DNA. |
| GI-4 | 17670 | tyrosine-type recombinase/integrase | *Enterococcus villorum* | 9e-80 | 81.0 | 90.0 | WP010752070.1 | Cleave DNA substrates by a series of staggered cuts, during which the protein becomes covalently linked to the DNA through a catalytic tyrosine residue at the carboxy end of the alignment. |
| GI-4 | 17700 | Response regulator | *Enterococcus hirae* | 6e-70 | 100.0 | 82.2 | EGP5406881.1 | DNA binding, and regulation of transcription. |
| GI-4 | 17720 | GHKL domain-containing protein | *Enterococcus villorum* | 9e-85 | 99.0 | 81.0 | WP010752108.1 | DNA structure rearrangement, heat shock, signal transduction, and DNA mismatch repair |
| GI-4 | 17730 | GHKL domain-containing protein | *Enterococcus villorum* | 1e-85 | 99.0 | 67.5 | WP010752108.1 |  |
| GI-5 | 24350 | FAD-dependant oxidoreductase | *Enterococcus termitis* | 0.0 | 99.0 | 59.0 | WP069662575.1 | Oxidoreductase activity and regulating the intracellular levels of amines via there oxidation; these include various neurotransmitters, neurotoxins and trace amines. |
| GI-5 | 24370 | M28 family peptidase | *Enterococcus hirae* | 5e-95 | 100.0 | 65.0 | WP193797416.1 | Metabolic and signalling pathways, and hydrolysis of peptides. |
| GI-5 | 24400 | Recombinase family protein | *Enterococcus faecium* | 2e-163 | 98.0 | 94.2 | WP129236197.1 | Site-specific recombination of DNA molecules by a concerted, four-strand cleavage and rejoining mechanism. |
| GI-5 | 24410 | Recombinase family protein | *Enterococcus durans* | 8e-55 | 100.0 | 95.5 | RGW56610.1 |  |

**Supplementary Table 7:** Tandem repeats identified in *E. lacertideformus*.

| **Indices** | **Period size** | **Copy number** | **Consensus size** | **Matches (%)** | **Indels (%)** | **Score** | **A** | **C** | **G** | **T** | **Entropy (0-2)** | **Protein-coding** |
| --- | --- | --- | --- | --- | --- | --- | --- | --- | --- | --- | --- | --- |
| **Contig 1** | | | | | | | | | | | |  |
| 7843-7887 | 21 | 2.1 | 21 | 87 | 0 | 63 | 22 | 26 | 11 | 40 | 1.87 | Y |
| 9902-9949 | 25 | 1.9 | 25 | 83 | 8 | 62 | 10 | 20 | 4 | 64 | 1.41 | N |
| 35279-35307 | 15 | 2.0 | 15 | 93 | 6 | 51 | 34 | 0 | 3 | 62 | 1.12 | N |
| **Contig 2** |  |  |  |  |  |  |  |  |  |  |  |  |
| 103978-104034 | 24 | 2.4 | 24 | 80 | 11 | 71 | 63 | 8 | 14 | 14 | 1.52 | Y |
| 105395-105422 | 12 | 2.3 | 12 | 100 | 0 | 56 | 67 | 7 | 17 | 7 | 1.37 | Y |
| **Contig 3** | | | | | | | | | | | |  |
| 164978-165342 | 195 | 1.9 | 195 | 97 | 0 | 685 | 48 | 16 | 20 | 15 | 1.81 | Y |
| 165374-166152 | 270 | 2.9 | 269 | 91 | 0 | 1168 | 38 | 13 | 22 | 24 | 1.91 | Y |
| 174964-175018 | 1 | 55.0 | 1 | 100 | 0 | 110 | 100 | 0 | 0 | 0 | 0.00 | N |
| 211191-211215 | 12 | 2.1 | 12 | 100 | 0 | 50 | 16 | 28 | 8 | 48 | 1.74 | Y |
| 216000-216059 | 12 | 5.0 | 12 | 100 | 0 | 120 | 16 | 16 | 8 | 58 | 1.61 | Y |
| **Contig 3/4** |  |  |  |  |  |  |  |  |  |  |  |  |
| 216239-216314 | 1 | 76.0 | 1 | 89 | 0 | 107 | 2 | 1 | 2 | 93 | 0.45 | N |
| **Contig 4** |  |  |  |  |  |  |  |  |  |  |  |  |
| 232489-232521 | 17 | 2.0 | 16 | 94 | 5 | 57 | 6 | 3 | 6 | 84 | 0.84 | N |
| 269886-269921 | 9 | 4.0 | 9 | 96 | 0 | 63 | 13 | 0 | 33 | 52 | 1.41 | Y |
| **Contig 4/5** |  |  |  |  |  |  |  |  |  |  |  |  |
| 273279-273773 | 42 | 12.4 | 42 | 83 | 12 | 475 | 29 | 27 | 27 | 15 | 1.96 | N |
| 273307-273367 | 30 | 2.0 | 30 | 100 | 0 | 122 | 31 | 22 | 29 | 16 | 1.96 | N |
| 273421-273481 | 30 | 2.0 | 30 | 100 | 0 | 122 | 31 | 22 | 29 | 16 | 1.96 | N |
| 273379-273605 | 72 | 3.0 | 72 | 87 | 12 | 364 | 30 | 27 | 28 | 13 | 1.94 | N |
| 273279-273676 | 114 | 3.4 | 114 | 87 | 7 | 527 | 29 | 29 | 27 | 14 | 1.95 | N |
| 273441-273605 | 82 | 2.0 | 82 | 100 | 0 | 330 | 32 | 25 | 29 | 13 | 1.93 | N |
| **Contig 5** |  |  |  |  |  |  |  |  |  |  |  |  |
| 273746-273833 | 30 | 2.9 | 30 | 98 | 0 | 167 | 31 | 20 | 30 | 17 | 1.95 | N |
| 273803-273883 | 42 | 1.9 | 42 | 94 | 0 | 144 | 33 | 28 | 25 | 12 | 1.92 | N |
| 273909-274152 | 12 | 20.8 | 12 | 69 | 9 | 119 | 53 | 8 | 22 | 15 | 1.69 | N |
| 273927-274216 | 36 | 8.1 | 35 | 79 | 13 | 200 | 54 | 7 | 22 | 15 | 1.67 | N |
| 273909-273996 | 24 | 3.7 | 24 | 92 | 3 | 133 | 53 | 9 | 23 | 13 | 1.68 | N |
| 273972-274219 | 18 | 13.4 | 18 | 81 | 10 | 261 | 53 | 7 | 21 | 17 | 1.67 | N |
| 274221-274255 | 18 | 1.9 | 18 | 88 | 0 | 52 | 68 | 8 | 5 | 17 | 1.35 | N |
| 274245-274307 | 12 | 5.3 | 12 | 96 | 0 | 117 | 65 | 0 | 9 | 25 | 1.23 | N |
| 274388-274464 | 33 | 2.3 | 34 | 82 | 13 | 106 | 49 | 10 | 24 | 15 | 1.76 | N |
| 283065-283407 | 177 | 1.9 | 177 | 84 | 3 | 458 | 21 | 18 | 19 | 41 | 1.91 | Y |
| 283659-283687 | 11 | 2.6 | 11 | 100 | 0 | 58 | 34 | 41 | 6 | 17 | 1.76 | Y |
| 308076-308163 | 42 | 2.1 | 42 | 100 | 0 | 176 | 19 | 12 | 20 | 47 | 1.81 | Y |
| 346736-346774 | 10 | 3.9 | 10 | 100 | 0 | 78 | 71 | 0 | 10 | 17 | 1.12 | Y |
| 375735-375764 | 15 | 2.0 | 15 | 100 | 0 | 60 | 33 | 33 | 13 | 20 | 1.91 | Y |
| 391941-391973 | 14 | 2.3 | 15 | 89 | 10 | 59 | 45 | 12 | 6 | 36 | 1.66 | Y |
| **Contig 7** |  |  |  |  |  |  |  |  |  |  |  |  |
| 449208-449241 | 17 | 2.0 | 17 | 100 | 0 | 68 | 41 | 17 | 5 | 35 | 1.74 | N |
| **Contig 7/8** |  |  |  |  |  |  |  |  |  |  |  |  |
| 465575-465822 | 124 | 2.0 | 124 | 100 | 0 | 496 | 25 | 16 | 21 | 36 | 1.94 | N |
| **Contig 8** |  |  |  |  |  |  |  |  |  |  |  |  |
| 480847-480881 | 17 | 2.1 | 17 | 100 | 0 | 70 | 25 | 17 | 28 | 28 | 1.97 | N |
| 483783-483815 | 9 | 3.7 | 9 | 95 | 0 | 57 | 30 | 45 | 24 | 0 | 1.53 | Y |
| 486095-486139 | 18 | 2.5 | 18 | 100 | 0 | 90 | 20 | 15 | 17 | 46 | 1.84 | Y |
| 561943-561989 | 24 | 2.0 | 24 | 82 | 0 | 58 | 14 | 17 | 17 | 51 | 1.77 | Y |
| 577703-577727 | 11 | 2.3 | 11 | 100 | 0 | 50 | 44 | 12 | 8 | 36 | 1.71 | Y |
| 579638-579663 | 8 | 3.3 | 8 | 100 | 0 | 52 | 53 | 0 | 0 | 46 | 1 | Y |
| 595182-595207 | 12 | 2.2 | 12 | 100 | 0 | 52 | 61 | 7 | 11 | 19 | 1.53 | N |
| **Contig 9** |  |  |  |  |  |  |  |  |  |  |  |  |
| 646197-646346 | 57 | 2.6 | 57 | 89 | 2 | 239 | 42 | 20 | 22 | 15 | 1.88 | Y |
| **Contig 10** |  |  |  |  |  |  |  |  |  |  |  |  |
| 750078-750105 | 11 | 2.5 | 11 | 100 | 0 | 56 | 0 | 0 | 35 | 64 | 0.94 | N |
| 753992-754023 | 12 | 2.7 | 12 | 90 | 0 | 55 | 56 | 25 | 12 | 6 | 1.59 | Y |
| 754066-754151 | 39 | 2.2 | 39 | 93 | 0 | 145 | 56 | 18 | 11 | 12 | 1.65 | Y |
| 815602-815641 | 16 | 2.5 | 16 | 100 | 0 | 80 | 42 | 15 | 15 | 27 | 1.86 | Y |
| **Contig 10/11** |  |  |  |  |  |  |  |  |  |  |  |  |
| 855131-855402 | 135 | 2.0 | 135 | 100 | 0 | 544 | 33 | 9 | 22 | 34 | 1.87 | N |
| **Contig 13** |  |  |  |  |  |  |  |  |  |  |  |  |
| 868585-868623 | 16 | 2.4 | 16 | 100 | 0 | 78 | 48 | 17 | 0 | 33 | 1.48 | N |
| 884576-884600 | 12 | 2.1 | 12 | 100 | 0 | 50 | 32 | 8 | 24 | 36 | 1.84 | Y |
| 901687-901715 | 9 | 3.2 | 9 | 100 | 0 | 58 | 65 | 10 | 10 | 13 | 1.47 | Y |
| 944501-944538 | 18 | 2.1 | 18 | 100 | 0 | 76 | 26 | 5 | 10 | 57 | 1.53 | Y |
| 946529-946798 | 124 | 2.2 | 124 | 97 | 0 | 504 | 37 | 14 | 21 | 27 | 1.91 | N |
| 965272-965299 | 10 | 2.8 | 10 | 100 | 0 | 56 | 10 | 7 | 0 | 82 | 0.85 | N |
| **Contig 14** |  |  |  |  |  |  |  |  |  |  |  |  |
| 1089603-1089637 | 12 | 2.9 | 12 | 91 | 0 | 52 | 62 | 14 | 8 | 14 | 1.53 | N |
| **Contig 15** |  |  |  |  |  |  |  |  |  |  |  |  |
| 1127973-1128012 | 21 | 2.0 | 21 | 85 | 14 | 57 | 35 | 15 | 15 | 35 | 1.88 | Y |
| 1191576-1191641 | 33 | 2.0 | 33 | 85 | 5 | 89 | 43 | 12 | 7 | 36 | 1.7 | Y |
| **Contig 16** |  |  |  |  |  |  |  |  |  |  |  |  |
| 1204902-1204935 | 17 | 1.9 | 17 | 88 | 5 | 50 | 50 | 5 | 5 | 38 | 1.51 | Y |
| 1220540-1220570 | 12 | 2.6 | 12 | 100 | 0 | 62 | 58 | 6 | 16 | 19 | 1.59 | N |
| 1220596-1220624 | 15 | 1.9 | 15 | 100 | 0 | 58 | 34 | 6 | 17 | 41 | 1.76 | N |
| 1232587-1232611 | 11 | 2.3 | 11 | 100 | 0 | 50 | 60 | 12 | 8 | 20 | 1.57 | Y |
| 1240358-1240405 | 24 | 2.0 | 24 | 87 | 0 | 69 | 14 | 22 | 16 | 45 | 1.84 | Y |
| 1314884-1314909 | 11 | 2.4 | 11 | 100 | 0 | 52 | 50 | 11 | 15 | 23 | 1.76 | N |
| 1318746-1318788 | 22 | 2.0 | 23 | 86 | 9 | 63 | 20 | 23 | 11 | 44 | 1.84 | Y |
| 1336923-1336969 | 24 | 2.0 | 22 | 92 | 8 | 76 | 8 | 8 | 8 | 74 | 1.22 | N |
| 1336923-1336969 | 11 | 4.1 | 11 | 86 | 10 | 67 | 8 | 8 | 8 | 74 | 1.22 | N |
| 1342762-1342787 | 8 | 3.3 | 8 | 100 | 0 | 52 | 0 | 34 | 0 | 65 | 0.93 | N |
| 1362877-1362905 | 15 | 1.9 | 15 | 100 | 0 | 58 | 20 | 37 | 6 | 34 | 1.8 | Y |
| 1365119-1365144 | 13 | 2.0 | 13 | 100 | 0 | 52 | 30 | 15 | 15 | 38 | 1.88 | N |
| 1387559-1387584 | 12 | 2.2 | 12 | 100 | 0 | 52 | 46 | 0 | 15 | 38 | 1.46 | Y |
| 1396727-1396766 | 21 | 1.9 | 21 | 89 | 0 | 62 | 15 | 12 | 40 | 32 | 1.84 | Y |
| 1409638-1409664 | 13 | 2.1 | 13 | 100 | 0 | 54 | 48 | 0 | 22 | 29 | 1.51 | Y |
| 1432293-1432346 | 28 | 1.9 | 28 | 85 | 7 | 74 | 16 | 7 | 20 | 55 | 1.65 | Y |
| 1455771-1455808 | 15 | 2.5 | 15 | 95 | 0 | 67 | 15 | 7 | 23 | 52 | 1.69 | N |
| 1469005-1469034 | 14 | 2.1 | 14 | 100 | 0 | 60 | 30 | 13 | 13 | 43 | 1.82 | Y |
| 1473924-1473967 | 16 | 2.8 | 16 | 100 | 0 | 88 | 52 | 18 | 4 | 25 | 1.64 | Y |
| 1513256-1514364 | 285 | 3.9 | 285 | 91 | 1 | 1631 | 20 | 25 | 18 | 35 | 1.95 | Y |
| 1557902-1557935 | 18 | 1.9 | 17 | 88 | 5 | 50 | 5 | 2 | 14 | 76 | 1.09 | Y |
| 1570266-1570337 | 21 | 3.4 | 21 | 100 | 0 | 144 | 18 | 8 | 9 | 63 | 1.48 | N |
| 1570280-1570337 | 12 | 5.3 | 12 | 69 | 23 | 56 | 17 | 10 | 8 | 63 | 1.49 | N |
| 1581332-1581389 | 25 | 2.3 | 25 | 96 | 0 | 107 | 13 | 22 | 13 | 50 | 1.77 | N |
| 1597073-1597106 | 13 | 2.6 | 13 | 100 | 0 | 68 | 67 | 0 | 20 | 11 | 1.21 | Y |
| 1608365-1608467 | 39 | 2.6 | 39 | 79 | 11 | 104 | 68 | 12 | 16 | 1 | 1.29 | Y |
| 1608411-1608475 | 21 | 3.2 | 21 | 79 | 14 | 75 | 70 | 12 | 15 | 1 | 1.23 | Y |
| **Contig 16/17** |  |  |  |  |  |  |  |  |  |  |  |  |
| 1627642-1627889 | 124 | 2.0 | 124 | 100 | 0 | 496 | 25 | 16 | 21 | 36 | 1.94 | N |
| **Contig 17** |  |  |  |  |  |  |  |  |  |  |  |  |
| 1635479-1635514 | 18 | 2.0 | 18 | 100 | 0 | 72 | 22 | 27 | 5 | 44 | 1.75 | Y |
| 1641790-1642319 | 207 | 2.6 | 207 | 92 | 1 | 857 | 37 | 22 | 15 | 24 | 1.93 | Y |
| 1658328-1658379 | 23 | 2.3 | 23 | 100 | 0 | 104 | 17 | 11 | 11 | 59 | 1.6 | Y |
| 1776679-1776709 | 15 | 2.1 | 15 | 93 | 0 | 53 | 25 | 25 | 12 | 35 | 1.92 | N |
| 1844031-1844064 | 17 | 1.9 | 18 | 88 | 5 | 52 | 50 | 32 | 5 | 11 | 1.63 | Y |
| 1874342-1874393 | 9 | 5.8 | 9 | 93 | 0 | 86 | 3 | 23 | 28 | 44 | 1.71 | Y |
| 1874337-1874396 | 18 | 3.3 | 18 | 90 | 0 | 93 | 6 | 25 | 26 | 41 | 1.8 | Y |
| 1874337-1874396 | 27 | 2.2 | 27 | 84 | 0 | 84 | 6 | 25 | 26 | 41 | 1.8 | Y |
| 1875788-1875945 | 42 | 3.8 | 42 | 89 | 0 | 226 | 22 | 18 | 24 | 34 | 1.96 | N |
| 1876262-1876319 | 23 | 2.5 | 22 | 77 | 5 | 62 | 56 | 12 | 0 | 31 | 1.35 | N |
| **Contig 18** |  |  |  |  |  |  |  |  |  |  |  |  |
| 1916261-1916307 | 21 | 2.3 | 21 | 85 | 3 | 60 | 48 | 2 | 14 | 34 | 1.56 | Y |
| **Contig 20** |  |  |  |  |  |  |  |  |  |  |  |  |
| 1985934-1985967 | 14 | 2.4 | 14 | 100 | 0 | 68 | 23 | 5 | 23 | 47 | 1.73 | Y |
| 1992963-1992992 | 13 | 2.3 | 13 | 100 | 0 | 60 | 30 | 33 | 6 | 30 | 1.83 | Y |
| 1997320-1997345 | 12 | 2.2 | 12 | 100 | 0 | 52 | 19 | 15 | 15 | 50 | 1.79 | Y |
| 2031299-2031383 | 39 | 2.2 | 39 | 100 | 0 | 170 | 52 | 23 | 11 | 11 | 1.7 | Y |
| 2042001-2042025 | 11 | 2.3 | 11 | 100 | 0 | 50 | 56 | 0 | 28 | 16 | 1.41 | Y |
| **Contig 22** |  |  |  |  |  |  |  |  |  |  |  |  |
| 2168157-2168183 | 9 | 3.0 | 9 | 100 | 0 | 54 | 33 | 11 | 11 | 44 | 1.75 | Y |
| 2190422-2190470 | 20 | 2.5 | 20 | 100 | 0 | 98 | 73 | 4 | 8 | 14 | 1.21 | N |
| 2215219-2215255 | 16 | 2.4 | 16 | 95 | 4 | 67 | 10 | 16 | 37 | 35 | 1.83 | Y |
| 2217140-2217177 | 18 | 2.1 | 19 | 90 | 5 | 60 | 23 | 18 | 5 | 52 | 1.65 | N |
| 2217242-2217276 | 17 | 2.0 | 18 | 88 | 5 | 54 | 48 | 25 | 2 | 22 | 1.64 | N |
| 2228401-2228442 | 21 | 2.0 | 20 | 90 | 4 | 66 | 64 | 7 | 7 | 21 | 1.43 | N |
| 2236305-2236368 | 29 | 2.2 | 29 | 100 | 0 | 128 | 45 | 15 | 17 | 21 | 1.85 | N |
| 2244515-2244581 | 29 | 2.3 | 29 | 97 | 0 | 125 | 52 | 5 | 17 | 23 | 1.67 | N |
| 2253377-2253401 | 12 | 2.1 | 12 | 100 | 0 | 50 | 40 | 28 | 8 | 24 | 1.83 | Y |
| **Contig 23** |  |  |  |  |  |  |  |  |  |  |  |  |
| 2277972-2278092 | 51 | 2.4 | 51 | 100 | 0 | 242 | 19 | 12 | 27 | 41 | 1.87 | Y |
| 2278127-2278198 | 33 | 2.2 | 33 | 84 | 0 | 99 | 26 | 8 | 27 | 37 | 1.85 | Y |
| 2312200-2312229 | 15 | 1.9 | 16 | 93 | 6 | 53 | 16 | 13 | 10 | 60 | 1.59 | Y |
| 2334814-2334854 | 13 | 3.2 | 13 | 100 | 0 | 82 | 17 | 7 | 14 | 60 | 1.55 | N |
| 2379109-2379156 | 18 | 2.7 | 18 | 90 | 0 | 78 | 12 | 10 | 18 | 58 | 1.62 | Y |
| 2380170-2380329 | 66 | 2.4 | 69 | 85 | 6 | 211 | 20 | 16 | 8 | 53 | 1.69 | Y |
| 2380175-2380360 | 69 | 2.8 | 68 | 84 | 4 | 216 | 20 | 16 | 8 | 54 | 1.68 | Y |

Period size, the most common matching distance between corresponding characters in the alignment/pattern size of the tandem repeat; copy number, the number of repetitions of a particular tandem repeat throughout the genome; consensus size, the size of the consensus sequence (may differ slightly from the consensus pattern); matches, percent of matches between adjacent copies overall; indels, percent of indels between adjacent copies overall; score, the alignment score for each repeat. This score must meet or exceed the minimum alignment score for the repeat to be reported; entropy, measure based on percent composition; protein-coding, indicates whether a particular tandem repeat occurs in or outside of a protein-coding gene.

**Supplementary Table 8:** Reference sequences included in the 16S rRNA and housekeeping MLST phylogenetic analyses.

| **Organism** | **Strain type** | **NCBI ID** |
| --- | --- | --- |
| *Enterococcus lacertideformus* | PHRS 0518 | JADAKE000000000. |
| *Enterococcus asini* | ATCC 700915 | ASVU01000000 |
| *Enterococcus avium* | 352 | CP034169 |
| *Enterococcus canis* | DSM 17029 | JXKH01000000 |
| *Enterococcus casseliflavus* | EC 291 | CP046123 |
| *Enterococcus cecorum* | NContigC 12422 | NZUGIY00000000 |
| *Enterococcus dispar* | ATCC 51266 | NZASWK00000000 |
| *Enterococcus durans* | BDGP3 | CP022930 |
| *Enterococcus faecalis* | OG1RF | NC017316 |
| *Enterococcus faecium* | AUS0085 | CP006620 |
| *Enterococcus faecium* | DO | CP003583 |
| *Enterococcus faecium* | NRRL B2354 | CP004063 |
| *Enterococcus gallinarum* | FDAARGOS 728 | CP046307 |
| *Enterococcus gilvus* | ATCC BAA 350 | ASWH01000000 |
| *Enterococcus haemoperoxidus* | ATCC BAA 382 | ASVY01000000 |
| *Enterococcus hirae* | R17 | NZCP015516 |
| *Enterococcus italicus* | DSM 15952 | GL622241 |
| *Enterococcus lactis* | CICC 24101 | NZWOTS01000000 |
| *Enterococcus malodoratus* | NContigC 12365 | NZUFXU01000000 |
| *Enterococcus mundtii* | DSM 4838 | CP018061 |
| *Enterococcus phoeniculicola* | ATCC BAA 412 | ASWE01000000 |
| *Enterococcus plantarum* | LMG 26214 | MIKA01000000 |
| *Enterococcus pseudoavium* | CBA 7133 | NNBZ01000000 |
| *Enterococcus raffinosus* | ATCC 49464 | ASWF01000000 |
| *Enterococcus ratti* | DSM 15687 | JXLB01000000 |
| *Enterococcus rotai* | LMG 26678 | CP013655 |
| *Enterococcus saccharolyticus* | 3012STDY6252241 | CAACXX010000000 |
| *Enterococcus sulfureus* | ATCC 49903 | KE136395 |
| *Enterococcus thailandicus* | A523 | CP023074 |
| *Enterococcus villorum* | F1129D | BJWF01000000 |
| *Enterococcus villorum* | NBRC 100699 | ASWG01000000 |
| *Vagococcus martis* | D7T301 | MVAB01000000 |
| *Vagococcus penaei* | CD276 | CP019609 |

**Supplementary Table 9:** *E. lacertideformus*-specific genes for each feature, and their position in the genome assembly.

| **Feature** | **COG ID** | **Gene** | **Name** | **Contig** | **Sequence start** | **Sequence end** |
| --- | --- | --- | --- | --- | --- | --- |
| C | COG1013 |  | Pyruvate-flavodoxin oxidoreductase | 16 | 1518588 | 1518752 |
| C | COG1013 |  | Pyruvate-flavodoxin oxidoreductase | 16 | 1518769 | 1519008 |
| C | COG1018 |  | FAD-dependent oxidoreductase | 22 | 2143268 | 2145502 |
| C | COG1390 | ntpE | V-type sodium ATPase subunit E | 17 | 1884432 | 1885016 |
| E | COG0686 |  | Alanine dehydrogenase | 15 | 1187200 | 1188309 |
| E | COG0747 |  | Hypothetical protein | 14 | 1089729 | 1090454 |
| G | COG1264 |  | PTS transporter subunit EIIB | 10 | 692867 | 693103 |
| G | COG1264 |  | PTS beta-glucoside transporter subunit EIIBCA | 3 | 157175 | 157543 |
| G | COG1264 |  | PTS glucose transporter subunit IIA | 17 | 1789511 | 1789792 |
| G | COG1264 |  | PTS beta-glucoside transporter subunit IIBCA | 14 | 1038736 | 1039173 |
| G | COG1626 |  | Alpha-glucosidase | 17 | 1643740 | 1645470 |
| I | COG0183 |  | Hydroxymethylglutaryl-CoA reductase, degradative | 14 | 997867 | 999075 |
| I | COG0615 |  | Glycerol-3-phosphate cytidylyltransferase | 13 | 867775 | 867972 |
| I | COG0615 |  | Glycerol-3-phosphate cytidylyltransferase | 13 | 868047 | 868169 |
| L | COG2189 |  | Site-specific DNA-methyltransferase | 22 | 2149014 | 2150231 |
| M | COG0615 |  | Glycerol-3-phosphate cytidylyltransferase | 13 | 867775 | 867972 |
| M | COG0615 |  | Glycerol-3-phosphate cytidylyltransferase | 13 | 868047 | 868169 |
| M | COG1519 |  | Glycosyltransferase | 17 | 1756221 | 1756412 |
| P | COG1840 |  | S-layer protein | 8 | 599233 | 599583 |
| P | COG3119 |  | Sulfatase | 14 | 1097170 | 1097451 |
| P | COG3119 |  | Sulfatase | 14 | 1096252 | 1097139 |
| P | COG3119 |  | Sulfatase | 14 | 1096052 | 1096252 |
| P | COG4535 |  | Hypothetical protein |  | 1306567 | 1307175 |
| Q | COG3473 |  | GNAT family N-acetyltransferase | 16 | 831356 | 831625 |
| R | COG0641 |  | SPASM domain-containing protein | 14 | 1097661 | 1098635 |
| R | COG0658 |  | DNA internalization-related competence protein ComEC/Rec2 | 15 | 1166501 | 1167421 |
| R | COG1205 |  | Hypothetical protein | 1 | 201 | 452 |
| R | COG2234 |  | M28 family peptidase | 22 | 2147610 | 2148254 |
| R | COG3393 |  | GNAT family N-acetyltransferase | 10 | 711505 | 712068 |
| S | COG1262 |  | Formylglycine-generating enzyme family protein | 14 | 1099424 | 1100275 |
| S | COG1944 |  | YcaO-like family protein | 10 | 733415 | 734737 |
| S | COG1944 |  | YcaO-like family protein | 10 | 727698 | 728744 |
| S | COG2327 |  | Polysaccharide pyruvyl transferase family protein | 14 | 978532 | 979722 |
| S | COG4199 |  | Single-stranded-DNA-specific exonuclease RecJ | 16 | 1249595 | 1249747 |
| S | COG4199 |  | Single-stranded-DNA-specific exonuclease RecJ | 16 | 1249707 | 1249970 |
| S | COG4922 |  | DUF5011 domain-containing protein | 4 | 247557 | 247772 |
| T | COG2204 |  | Sigma 54-interacting transcriptional regulator | 7 | 449964 | 450536 |

[C], Energy production & conversion; [E], Amino acid transport & metabolism; [G], Carbohydrate transport & metabolism; [H], Coenzyme transport & metabolism; [I], Lipid transport & metabolism; [L], Replication, recombination & repair; [M], Cell wall/membrane/envelope biogenesis; [P], Inorganic ion transport & metabolism; [Q], Secondary metabolites biosynthesis, transport & catabolism; [R], General function prediction only; [S], Function unknown; [T], Signal transduction mechanisms.

**Supplementary Table 10:** COG features present in comparator genomes *E. villorum, E. hirae, E. faecium* and *E. faecalis* and absent in *E. lacertideformus*.

| **Feature** | **COG ID** | **Name** |
| --- | --- | --- |
| C* | COG0543 | NAD(P)H-flavin reductase |
| C* | COG0674 | Pyruvate:ferredoxin oxidoreductase and related 2-oxoacid:ferredoxin oxidoreductases, alpha subunit. |
| C* | COG1141 | Ferredoxin |
| C* | COG1227 | Inorganic pyrophosphatase/exopolyphosphatase |
| C* | COG1252 | NADH dehydrogenase, FAD-containing subunit |
| E* | COG0136 | Aspartate-semialdehyde dehydrogenase |
| E* | COG0253 | Diaminopimelate epimerase |
| E* | COG0289 | Dihydrodipicolinate reductase |
| E* | COG0329 | Dihydrodipicolinate synthase/N-acetylneuraminate lyase |
| E* | COG0509 | Glycine cleavage system H protein (lipoate-binding) |
| E* | COG0527 | Aspartokinase |
| E* | COG0626 | Cystathionine beta-lyase/cystathionine gamma-synthase |
| E* | COG2171 | Tetrahydrodipicolinate N-succinyltransferase |
| E* | COG2423 | Ornithine cyclodeaminase/archaeal alanine dehydrogenase, mu-crystallin family |
| E* | COG2755 | Lysophospholipase L1 or related esterase |
| F* | COG0284 | Orotidine-5'-phosphate decarboxylase |
| F* | COG0461 | Orotate phosphoribosyltransferase |
| F* | COG1957 | Inosine-uridine nucleoside N-ribohydrolase |
| G* | COG1082 | Sugar phosphate isomerase/epimerase |
| G* | COG1554 | Trehalose and maltose hydrolase |
| G* | COG1929 | Glycerate kinase |
| G* | COG2182 | Maltose-binding periplasmic protein MalE |
| G* | COG2376 | Dihydroxyacetone kinase |
| G* | COG3833 | ABC-type maltose transport system, permease component |
| H* | COG0543 | NAD(P)H-flavin reductase |
| J | COG0257 | Ribosomal protein L36 |
| L | COG0675 | Transposase |
| L | COG2818 | 3-methyladenine DNA glycosylase Tag |
| M | COG0329 | Dihydrodipicolinate synthase/N-acetylneuraminate lyase |
| M | COG3049 | Penicillin V acylase or related amidase, Ntn superfamily |
| N | COG1989 | Prepilin signal peptidase PulO (type II secretory pathway) or related peptidase |
| O | COG1989 | Prepilin signal peptidase PulO (type II secretory pathway) or related peptidase |
| P* | COG0370 | Fe2+ transport system protein B |
| P* | COG1918 | Fe2+ transport system protein FeoA |
| R | COG0384 | Predicted epimerase YddE/YHI9, PhzF superfamily |
| R | COG1380 | Putative effector of murein hydrolase LrgA, UPF0299 family |
| R | COG1473 | Metal-dependent amidase/aminoacylase/carboxypeptidase |
| R | COG3568 | Metal-dependent hydrolase, endonuclease/exonuclease/phosphatase family |
| R | COG3607 | Predicted lactoylglutathione lyase |
| R | COG3654 | Prophage maintenance system killer protein |
| S | COG0759 | Membrane-anchored protein YidD, putatitve component of membrane protein insertase Oxa1/YidC/SpoIIIJ |
| S | COG3027 | Cell division protein ZapA, inhibits GTPase activity of FtsZ |
| S | COG3304 | Uncharacterized membrane protein YccF, DUF307 family |
| S | COG3412 | PTS-EIIA-like component DhaM of the dihydroxyacetone kinase DhaKLM complex |
| S | COG3759 | Uncharacterized membrane protein |
| S | COG4479 | Uncharacterized protein YozE, UPF0346 family |
| S | COG4698 | Uncharacterized protein YpmS, DUF2140 family |
| S | COG4703 | Uncharacterized protein YkuJ, DUF1797 family |
| S | COG4722 | Phage-related protein |
| S | COG4769 | Predicted membrane protein |
| S | COG5341 | Uncharacterized protein |
| T | COG3476 | Tryptophan-rich sensory protein |
| U | COG1989 | Prepilin signal peptidase PulO (type II secretory pathway) or related peptidase |
| V | COG4767 | Glycopeptide antibiotics resistance protein |

*COG features with metabolism functionality; [C], Energy production & conversion; [E], Amino acid transport & metabolism; [F], Nucleotide transport & metabolism; [G], Carbohydrate transport & metabolism; [H], Coenzyme transport & metabolism; [J], Translation, ribosomal structure & biogenesis; [L], Replication, recombination & repair; [M], Cell wall/membrane/envelope biogenesis; [N], Cell motility; [O], Posttranslational modification, protein turnover, chaperones; [P], Inorganic ion transport & metabolism; [R], General function prediction only; [S], Function unknown; [T], Signal transduction mechanisms; [U], Intracellular trafficking, secretion & vesicular transport; [V], Defence mechanisms.

**Supplementary Table 11:** Function and query data of [M] feature genes (cell wall/membrane/envelope biogenesis) identified in regions unique to *E. lacertideformus.* Region and Gene ID values are references referring to Figures 1 and 3.

| **Region ID** | **Gene ID** | **Operon ID** | **Gene** | **Function** | **Organism** | **Query/ identity (%)** | **E-value** | **NCBI accession** |
| --- | --- | --- | --- | --- | --- | --- | --- | --- |
| M-2 | a |  | Sugar transferase | LPS biosynthesis (colanic, teichoic acid) | *Paenibacillus protaetiae* | 98.0/58.7 | 1e-63 | WP129441600.1 |
|  | b | OP-1 | UDP-N-acetylglucosamine 2-epimerase | Enterobacterial common antigen biosynthesis and outer membrane biogenesis | *Enterococcus durans* | 87.0/55.5 | 9e-33 | STP28597.1 |
|  | c | OP-1 |  |  | *Enterococcus thailandicus* | 94.0/48.8 | 4e-18 | OJG93842.1 |
|  | d | OP-1 | LCP family protein | Anionic cell wall polymer biosynthesis enzyme, LytR-Cps2A-Psr (LCP) family | *Enterococcus ratti* | 98.0/56.9 | 2e-121 | WP071856207.1 |
|  | e |  | WecB/TagA/CpsF family glycosyltransferase | Biosynthesis of repeating polysaccharide units found in membrane glycolipids | *Enterococcus faecalis* | 80.0/61.7 | 1e-47 | WP163402740.1 |
|  | f |  | Polysaccharide export protein | Capsular polysaccharide biosynthesis protein | *Enterococcus hirae* | 85.0/49.4 | 1e-45 | WP063626450.1 |
|  | g |  | Glycosyltransferase family 4 protein | Glycosyltransferase involved in cell wall biosynthesis | *Enterococcus hirae* | 96.0/64.6 | 9e-96 | WP143716033.1 |
| M-3 | a | OP-2 | Sugar transferase | LPS biosynthesis (colanic, teichoic acid) | *Enterococcus villorum* | 95.0/62.8 | 7e-86 | WP010752256.1 |
|  | b |  | LCP family protein | Anionic cell wall polymer biosynthesis enzyme | *Enterococcus villorum* | 97.0/51.5 | 3e-101 | WP010750691.1 |
|  | c | OP-3 | Glycosyltransferase | Cell wall biosynthesis | *Enterococcus mundtii* | 98.0/65.3 | 2e-174 | WP086334335.1 |
|  | d | OP-3 | Polysaccharide export protein | Capsular polysaccharide biosynthesis protein | *Enterococcus mundtii* | 96.0/66.1 | 6e-84 | WP086334336.1 |
|  | e | OP-4 | Oligosaccharide flippase family protein | Export of O-antigen and teichoic acid and biosynthesis of peptidoglycan | *Bacillus anthracis* | 98.0/55.4 | 6e-175 | PGQ84490.1 |
|  | f | OP-4 | Glycosyltransferase | Cell wall biosynthesis | *Enterococcus faecium* | 96.0/36.8 | 6e-65 | WP098041095.1 |
|  | g | OP-5 | UDP-N-acetyl-D-glucosamine dehydrogenase | Catalyse the NAD-dependent alcohol-to-acid oxidation of nucleotide-linked sugars | *Enterococcus villorum* | 100.0/78.3 | 0.0 | OQO72235.1 |
|  | h | OP-5 | Aminotransferase class I/II-fold pyridoxal phosphate-dependent enzyme | Regulation of cell wall biogenesis | *Enterococcus villorum* | 100.0/78.3 | 3e-74 | WP010752244.1 |
|  | i | OP-5 |  |  | *Enterococcus villorum* | 100.0/70.9 | 1e-122 | WP010752244.1 |
|  | j | OP-5 | Glycosyltransferase | Cell wall biosynthesis | *Enterococcus mundtii* | 96.0/74.0 | 4e-41 | WP086334344.1 |
|  | k | OP-5 |  |  | *Enterococcus villorum* | 96.0/72.5 | 4e-56 | WP146811660.1 |
|  | l | OP-5 | Tyrosine protein kinase | Capsular polysaccharide biosynthesis and export of complex carbohydrates | *Enterococcus villorum* | 96.0/59.8 | 3e-107 | WP010752241.1 |
|  | m | OP-5 | Glycosyltransferase | Cell wall biosynthesis | *Enterococcus villorum* | 97.0/68.7 | 3e-179 | WP081205034.1 |
|  | n |  | Nucleotide sugar dehydrogenase | Capsular polysaccharide biosynthesis | *Enterococcus villorum* | 98.0/66.3 | 0.0 | WP081205035.1 |
|  | o | OP-6 | LPXTG cell wall anchor domain-containing protein | Promotes bacterial adhesion and cell wall attachment | *Enterococcus mundtii* | 99.0/50.6 | 7e-171 | WP161330666.1 |
|  | p | OP-6 |  |  | *Enterococcus phoeniclicola* | 99.0/49.8 | 4e-179 | WP169405747.1 |
| M-4 | a | OP-7 | Polysaccharide deacetylase | Catalyse the deacetylation of acetylated sugars on cell membranes | *Streptococcus mutans* | 96.0/42.5 | 8e-83 | WP019313892.1 |
| M-5 | a | OP-8 | Tyrosine protein kinase | Capsular polysaccharide biosynthesis and export of complex carbohydrates | *Enterococcus villorum* | 100.0/89.3 | 5e-152 | WP010750632.1 |
|  | b | OP-9 | Class D sortase | Anchor surface proteins to peptidoglycans of the bacterial cell wall envelope | *Enterococcus villorum* | 94.0/85.6 | 5e-134 | WP081183575.1 |
|  | c | OP-10 | Tyrosine protein phosphatase | Capsular polysaccharide biosynthesis protein | *Enterococcus villorum* | 100.0/83.1 | 3e-157 | WP081183571.1 |
|  | d | OP-10 | Sugar transferase | LPS biosynthesis (colanic, teichoic acid) | *Enterococcus villorum* | 100.0/90.4 | 8e-151 | WP010750625.1 |
|  | e | OP-10 | Glycosyltransferase | Cell wall biosynthesis | *Enterococcus villorum* | 100.0/80.9 | 0.0 | WP081183570.1 |
|  | f | OP-11 |  |  |  | 99.0/77.9 | 0.0 | WP010750619.1 |
|  | g | OP-11 | NAD(P)-dependent oxidoreductase | Cell wall biogenesis | *Enterococcus villorum* | 97.0/89.9 | 0.0 | WP010750618.1 |
|  | h | OP-11 | Nucleotide sugar dehydrogenase | Capsular polysaccharide biosynthesis | *Enterococcus villorum* | 100.0/80.7 | 0.0 | WP010750617.1 |
|  | i | OP-11 | Oligosaccharide flippase family protein | Membrane protein involved in export of O-antigen and teichoic acid and biosynthesis of peptidoglycan | *Enterococcus ratti* | 89.0/86.3 | 0.0 | WP071855404.1 |
| M-7 | a | OP-12 | SIS domain-containing protein | Cell envelope biosynthesis | *Enterococcus durans* | 100.0/88.2 | 0.0 | WP115234715.1 |
| M-10 | a | OP-13 | Sugar transferase | LPS biosynthesis (colanic, teichoic acid) | *Enterococcus faecium* | 81.3/88.9 | 2e-18 | KWY24998.1 |
|  | b | OP-13 | Glycosyltransferase family 1 protein | Capsular polysaccharide biosynthesis | *Enterococcus hirae* | 100.0/90.3 | 0.0 | WP123875035.1 |
|  | c | OP-13 | CDP-glycerol glycerophosphotransferase family protein | Polymerisation of the main chain of the teichoic acid | *Enterococcus hirae* | 95.0/87.6 | 2e-55 | WP123875031.1 |
|  | d | OP-13 |  |  |  | 100.0/85.8 | 1e-166 | WP151346311.1 |
|  | e | OP-13 | Oligosaccharide flippase family protein | Membrane protein involved in export of O-antigen and teichoic acid and biosynthesis of peptidoglycan | *Enterococcus hirae* | 100.0/93.6 | 0.0 | WP088745100.1 |
|  | f | OP-13 | Glycerol-3-phosphate cytidylyltransferase | Teichoic acid biosynthesis | *Enterococcus hirae* | 93.0/78.7 | 8e-26 | WP123875032.1 |
|  | g |  |  |  |  | 100.0/95.0 | 7e-19 | WP123875032.1 |
| M-15 | a |  | D-alanine-D-alanine carboxypeptidase | Bacterial cell wall biosynthesis | *Streptococcus agalactiae* | 98.0/52.8 | 1e-86 | AIK73321.1 |
| M-17 | a |  | Class C sortase | Pili assembly, microbial adhesion promotion, and biofilm formation | *Enterococcus massiliensis* | 99.0/67.4 | 2e-141 | WP048603356.1 |
|  | b |  | VWA domain-containing protein | Cell adhesion and synthesis of ECM proteins | *Enterococcus mediterraneensis* | 82.0/57.3 | 1e-49 | WP122646097.1 |
|  | c |  |  |  | *Enterococcus viikkiensis* | 94.0/43.8 | 5e-21 | WP137613993.1 |
|  | d |  | Peptidase P60 | Hydrolysis of cell wall polysaccharides | *Enterococcus faecalis* | 100.0/95.4 | 0.0 | ETC92020.1 |
|  | e |  | Cna protein B-type domain protein | Collagen binding surface protein | *Enterococcus faecium* | 99.0/89.4 | 5e-110 | EJY27481.1 |
|  | f |  |  |  |  | 98.0/94.0 | 4e-70 | EJY00312.1 |
|  | g |  | Fibrinogen-binding MSCRAMM adhesin Fss3 | Bind to fibrinogen and adhere to host cells to initiate infection | *Enterococcus faecium* | 93.0/94.2 | 0.0 | WP002335941.1 |
| M-18 | a |  | glycosyltransferase family 2 protein | Cell wall biosynthesis | *Enterococcus faecium* | 97.0/77.4 | 1e-166 | WP002345238.1 |
|  | b |  | UDP-N-acetylglucosamine 2-epimerase (non-hydrolysing) | Cell envelope, Biosynthesis and degradation of surface polysaccharides and lipopolysaccharides | *Enterococcus durans* | 100.0/90.4 | 0.0 | WP142422928.1 |
|  | c |  | Capsular polysaccharide biosynthesis protein | Capsular polysaccharide biosynthesis and modification of cell wall carbohydrates | *Enterococcus faecalis* | 100.0/89.7 | 7e-71 | KII51063.1 |
|  | d |  | Capsular polysaccharide biosynthesis protein CapF | Capsular polysaccharide assembling protein and cell envelope, biosynthesis and degradation of surface polysaccharides and lipopolysaccharides | *Enterococcus faecium* | 100.0/79.3 | 3e-107 | WP113808585.1 |
|  | e |  | Polysaccharide biosynthesis protein | Capsular polysaccharide biosynthesis protein | *Enterococcus faecium* | 100.0/90.6 | 0.0 | WP043952237.1 |
|  | f |  | Glycosyltransferase | Cell wall biosynthesis | *Enterococcus faecium* | 84.0/67.9 | 2e-16 | WP125190870.1 |
|  | g |  | Sugar transferase | LPS biosynthesis (colanic, teichoic acid) | *Enterococcus villorum* | 98.0/77.0 | 1e-33 | WP035007644.1 |

ECM, extracellular matrix; LPS, lipopolysaccharide.
